# Experimental Pathways towards Developing a Rotavirus Reverse Genetics System: Synthetic Full Length Rotavirus ssRNAs Are Neither Infectious nor Translated in Permissive Cells

**DOI:** 10.1371/journal.pone.0074328

**Published:** 2013-09-03

**Authors:** James E. Richards, Ulrich Desselberger, Andrew M. Lever

**Affiliations:** Department of Medicine, University of Cambridge, Addenbrooke’s Hospital, Cambridge, United Kingdom; Kantonal Hospital St. Gallen, Switzerland

## Abstract

At present the ability to create rationally engineered mutant rotaviruses is limited because of the lack of a tractable helper virus-free reverse genetics system. Using the cell culture adapted bovine RV RF strain (G6P6 [1]), we have attempted to recover infectious RV by co-transfecting *in vitro* transcribed ssRNAs which are identical in sequence to the positive sense strand of each of the 11 dsRNA genomic segments of the RF strain. The RNAs were produced either from cDNAs cloned by a target sequence-independent procedure, or from purified double layered RV particles (DLPs). We have validated their translational function by *in vitro* synthesis of ^35^S-labelled proteins in rabbit reticulocyte lysates; all 11 proteins encoded by the RV genome were expressed. Transfection experiments with DLP- or cDNA-derived ssRNAs suggested that the RNAs do not act independently as mRNAs for protein synthesis, once delivered into various mammalian cell lines, and exhibit cytotoxicity. Transfected RNAs were not infectious since a viral cytopathic effect was not observed after infection of MA104 cells with lysates from transfected cells. By contrast, an engineered mRNA encoding eGFP was expressed when transfected under identical conditions into the same cell lines. Co-expression of plasmids encoding NSP2 and NSP5 using a fowlpox T7 polymerase recombinant virus revealed viroplasm-like structure formation, but this did not enable the translation of transfected RV ssRNAs. Attempts to recover RV from ssRNAs transcribed intracellularly from transfected cDNAs were also unsuccessful and suggested that these RNAs were also not translated, in contrast to successful translation from a transfected cDNA encoding an eGFP mRNA.

## Introduction

Rotaviruses (RVs) infect the young of a large variety of mammalian and avian species [1] and are a major cause of acute gastroenteritis in infants and young children worldwide. They cause more than half a million deaths per annum, mainly in sub-Saharan Africa and South East Asia [2]. Since 2006, two live attenuated RV vaccines [3], [4] have been licensed in many countries and are being widely used, often in universal mass vaccination programs. In 2009, the WHO recommended their worldwide application [5]. Vaccination has led to a significant decrease in hospitalisation of infants for RV-associated acute gastroenteritis [6], [7], [8] yet their overall effectiveness is under scrutiny [9], [10], [11], [12].

Rotaviruses form a genus of the *Reoviridae* family and possess a genome consisting of 11 segments of double-stranded (ds) RNA encoding 6 structural and 6 non-structural proteins [1]. RVs are genomically and antigenically diverse, being classified into 7–8 species and recently (mainly species A) into RNA segment based genotypes [13], [14]. Like all RNA viruses, RVs have a high mutation rate [15], [16]. The genomes evolve through the mechanisms of sequential point mutation, genome reassortment, gene rearrangement, true genetic recombination, and (for human RV infections) zoonotic transmission [17]. There is extensive published research into the replication cycle and molecular pathogenesis [1], but precise correlation between genotype and phenotype requires engineered mutations of individual segments on a stable genetic background. The technology to generate these genetically defined viruses consists of reverse transcription of RV RNAs into cDNAs, mutagenesis at the cDNA level, re-transcription of ssRNA from cDNAs and incorporation of the mutated genes into viable infectious viral progeny (virus rescue), a process termed ‘reverse genetics’ (RG). Ideally RG systems do not depend on helper viruses since separation of an engineered virus from an excess of helper virus may be difficult, often requiring powerful specific selection mechanisms. For other viruses of the *Reoviridae* such as orthoreoviruses [18], [19] and orbiviruses [20], [21] helper virus-independent, plasmid only-based RG systems have been established. For RVs only helper virus-dependent systems have as yet been developed; recently with the ability to stably incorporate heterogeneous RNA sequences [22], [23], [24], [25], [26].

Here we describe attempts to develop a helper virus-free RG system for RVs by technologies successfully used in related RNA viruses [20], . Our attempts have so far been unsuccessful in rescuing infectious virus. However, we believe that presentation of our data which identifies the significant stumbling block of efficient protein expression from both RV cDNA and ssRNA, despite using a combination of techniques, will be valuable for the understanding of RV molecular biology. Additionally, the conclusions derived from our data will assist the continued efforts to develop a helper virus-free RG system and to further dissect the intracellular mechanism by which RV ssRNAs are translated.

## Results

### Full Length Amplification of cDNA (FLAC) from RV RF Genomic Template and Cloning of RV cDNAs

Applying full length amplification of cDNA (FLAC) technology [30], which has been used to synthesise cDNAs of several members of the *Reoviridae* [20], [31], we successfully amplified all 11 segments (Figure S1). The TOPO-TA pCR2.1 cloning kit (Invitrogen) was used to shotgun-clone these products of the FLAC reaction. Numerous clones were sequenced (Table S1) and used to generate a consensus sequence for each segment (Table S2), applying cogent GenBank sequences as a guide (Table S3). All cDNA clone-derived sequences for each segment were aligned (including the GenBank data) using ClustalX2 (unpublished data). Full sequences of each segment used for the respective alignments and consensus sequences can be found in GenBank (for accession numbers see Tables S1 & S2). Individual clones possessing the consensus sequence were used as templates for PCR to synthesize transcriptional cassettes (Figure S2).

### *In silico* Analysis of Consensus RV Sequences

Compared to the GenBank RF strain the consensus sequences for segment 1, 6 and 9 were identical whereas segments 2–5 and 7, 8, 10 & 11 had point mutations. Segments 1 and 6 were used for protein translation studies. We examined *in silico* the impact these mutations might have on RNA structure to ensure that no significant conformational changes were present which could impair efficient translation. An extensive *in silico* analysis which incorporated all available full length RV species A sequences for each of the 11 segments identified potential stable long range *cis*-acting interactions (LRI) between the 5′ and 3′ ends of almost all segments [32]. Using RNAfold we compared each segment where mutations had arisen to the structure produced from the GenBank sequence. Figure S3 shows that the mutations had minimal effects on the terminal RNA structures. Segments 2 and 7 showed the greatest alteration in RNA structure but this only occurred at the extreme termini of the RNA whilst the majority of the terminal structure remained intact. Further comparison of our sequences with the ConStruct data from Li *et al*., was unnecessary for segments 1, 6 and 9 as there were no mutations between our consensus and the GenBank sequence. For segments 8 and 11 no predicted alterations to the RNA structures were found. Segment 8 encoded two mutations (N#63 & 64) which were a reversal of nucleotides GC to CG in a stable stem structure. We also noted that our segment 11 consensus sequence, which encoded an additional U residue (N#4) was consistent with the consensus sequence published by Li *et al*. [32].

### Transcription *in vitro* of RV ssRNA from Recombinant pUC19 Clones

**Figure 1:**
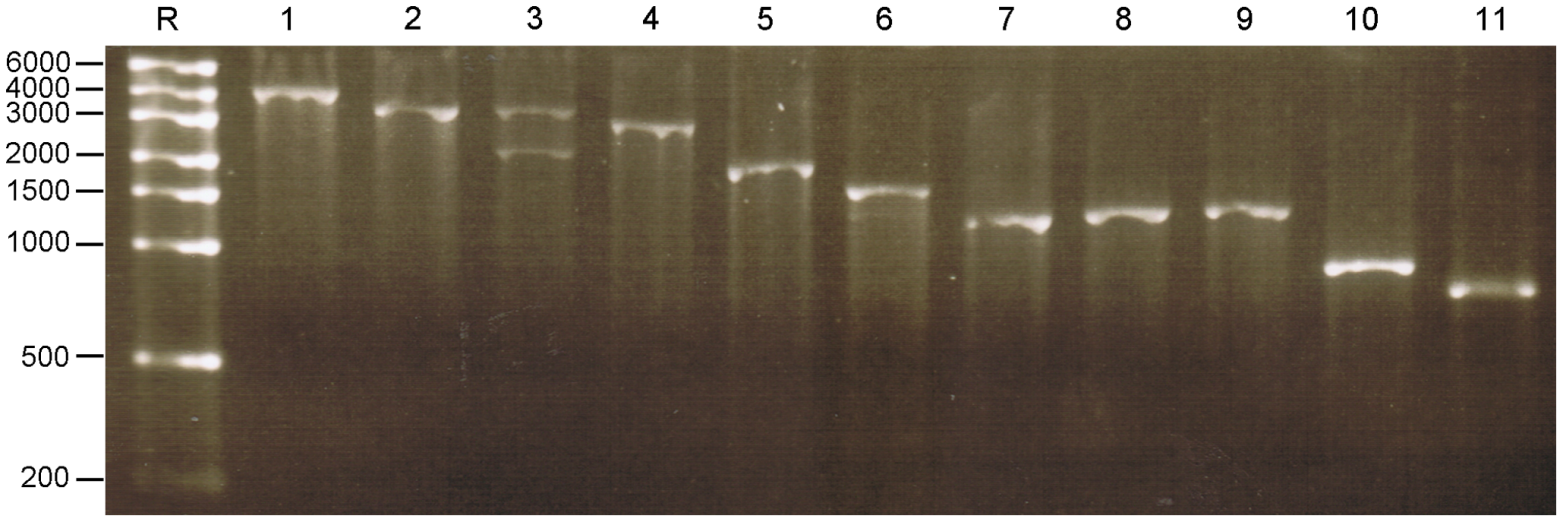
Agarose gel electrophoresis of the *in vitro* transcribed ssRNAs of the 11 RF RV segments. *In vitro* transcribed viral positive sense RNA, transcribed in the presence of a cap analogue from linear templates with a RE digested 3′ end. 200 ng of each ssRNA transcript was loaded onto the gel; 1.5% TBE AGE 80 V for 45 min. Lane R: RiboRuler™ High Range RNA markers (in bases); lanes 1–11: positive sense co-capped RV RF ssRNAs corresponding to segments 1 to 11, respectively.

Transcription templates were digested with the appropriate restriction enzyme (RE) to define the 3′ end of the RV segment and then purified prior to *in vitro* transcription with T7 Pol. The resulting ssRNA transcripts were analysed by TBE-AGE as shown in Figure 1. Nine of 11 templates produced a unique ssRNA band of the appropriate size. Transcription products of segment 3 and 6 cDNA templates each migrated as double bands, one of the expected full length ssRNA and a second smaller transcript. The same dual product was seen for both segments when PCR amplicons were used as transcription templates (unpublished data), strongly suggesting that the shorter ssRNA had resulted from premature termination of transcription [33]. To ensure the bands were not gel-related artifacts, samples were separated by denaturing urea PAGE, and the same profile was seen (unpublished data). BLAST analyses did not detect intragenic sequences similar to the T7 Pol promoter or terminator sequences of segment 3 and segment 6 (unpublished data).

### ssRNA Synthesis from Purified DLPs of Several RV Strains

**Figure 2:**
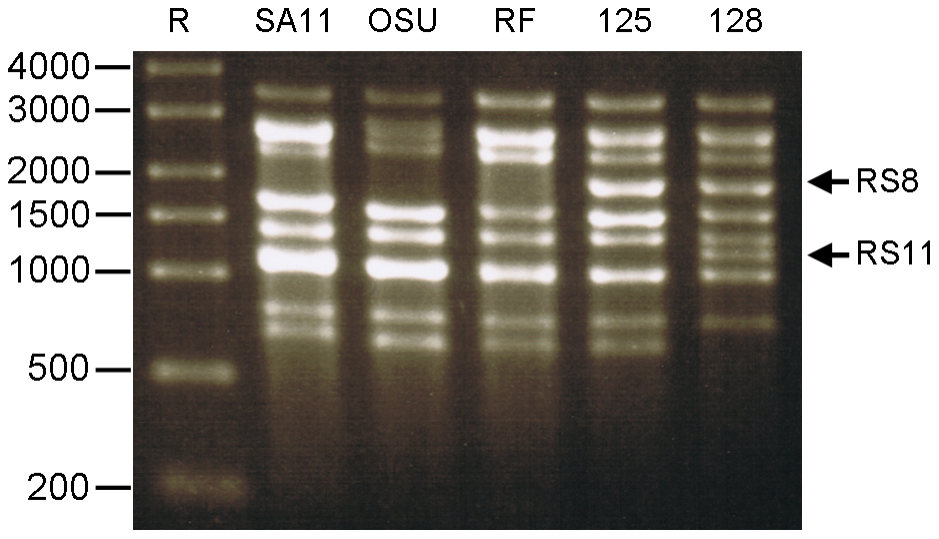
ssRNA derived from several strains of RV DLPs. ssRNA were synthesized *in vitro* from purified RV DLPs. DLPs were incubated at 37°C for 2 hours with the necessary components for positive sense ssRNA synthesis. 1.5% TBE AGE 45 V for 120 min. Lane R: RiboRuler™ High Range (in bases); 1.5 µg ssRNA derived from DLPs of RV strains SA11, OSU, RF, 125 and 128, respectively. DLP-derived ssRNAs 2 and 3 comigrate in all samples., ssRNAS 7–9 comigrate in SA11, OSU and RF, but only ssRNAS 7 and 9 comigrate in 125 and 128. Arrows indicate the altered migration of ssRNAs synthesised from the templates of rearranged genomic segments (RS). RS8, RS11 rearranged segment 8 or 11, respectively, of RV strains 125 and 128.

Purified RV DLPs can transcribe positive ssRNA *in vitro* from the dsRNA genomic segments packaged in the particles [34], [35], [36]. RNAs extruding from DLPs are positive sense and should possess authentic *in vivo* structures to facilitate experiments aimed at recovering infectious RV. DLPs of five different RV strains were used; the yields of ssRNA were between 3 and 10 µg of purified ssRNA per µg of purified DLPs. Figure 2 shows the successful synthesis of ssRNAs from the DLPs of all strains used alongside a ssRNA marker. The AGE migration patterns in Figure 2, lanes 2–6 reveal the genome structure of each strain. Thus, it was possible to identify ssRNAs derived from DLPs containing rearranged genomes (lanes 4 and 5).

### *In vitro* Synthesis of an eGFP-encoding mRNA

**Figure 3:**
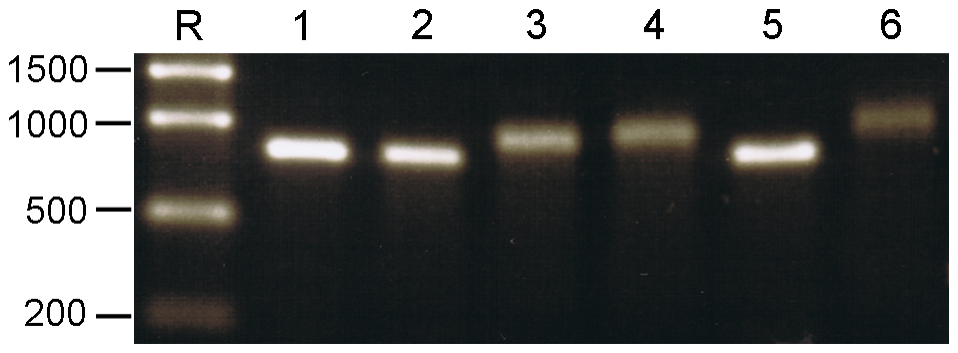
eGFP ssRNA species produced *in vitro*. ssRNAs were synthesised from PCR-derived amplicons with a T7 Pol promoter introduced at the 5′ end to facilitate *in vitro* transcription. PCR amplicons were digested with *Bsm*BI to define the 3′ end of the transcription cassette. Templates, either 500 ng or 1 µg, were incubated with T7 Pol in the absence or presence of a cap analogue using MEGAscript® or Mmessage Mmachine®. Uncapped ssRNAs were purified and post-transcriptionally capped using ScriptCap™ m7G Capping System. ssRNA was polyadenylated using *E. coli* polyadenylation polymerase (ePAP) Ambion. Lane R: RiboRuler™ High Range; lanes 1–6: approximately 250 ng each of eGFP ssRNA; uncapped, post-capped, uncapped polyadenylated, post-capped polyadenylated, co-capped, co-capped polyadenylated, respectively. 1.5% TBE AGE 60 V for –90 min. The polyadenylated RNA bands are less sharp as the molecules differ in the numbers of A residues added at the 3′end.

We used a commercial vector (pEGFP-N1; Clontech) to create an *in vitro* transcribed mRNA so that the autofluorescence of expressed enhanced GFP (eGFP) could be used as a marker for the efficiency of RNA transfection into mammalian cells. The requirement for a reporter ssRNA relying on conventional mammalian translation machinery rather than utilising RV UTRs was essential as it has been shown that a transfected luciferase reporter gene flanked with the UTR of gene 6 was only expressed efficiently in cells already infected with RV [37]. Furthermore a recent publication has demonstrated that ssRNA transfection of a chimeric mRNA also utilising RV segment 6 UTRs to control translation was less efficient than a globin control [38]. Using similar technology to that described above, the eGFP ORF was amplified using PCR with primers which introduced a T7 Pol promoter fused to the 5′ end of the transcriptional start point and a *Bsm*BI site to define the 3′ end. *Bsm*BI-digested T7 eGFP amplicons were used as a template for *in* *vitro* transcription as described, and transcripts were additionally polyadenylated using *E. coli* poly (A) polymerase (Ambion) to resemble mammalian mRNAs. Samples of uncapped, post-transcriptionally capped (‘post-capped’) and co-transcriptionally capped (‘co-capped’) eGFP ssRNA were analysed by AGE (Figure 3, lanes 1, 2 and 5, respectively). Samples of uncapped, post-capped and co-capped polyadenylated eGFP ssRNAs were also analysed (Figure 3, lanes 3, 4 and 6, respectively). Figure 3 shows that uncapped, post-capped and co-capped ssRNAs all migrate at the same rate and that the three respective polyadenylated RNA species also co-migrated, although at an expected slower rate.

### *In vitro* Translation of Proteins from *in vitro* Transcribed RV RNA

**Figure 4:**
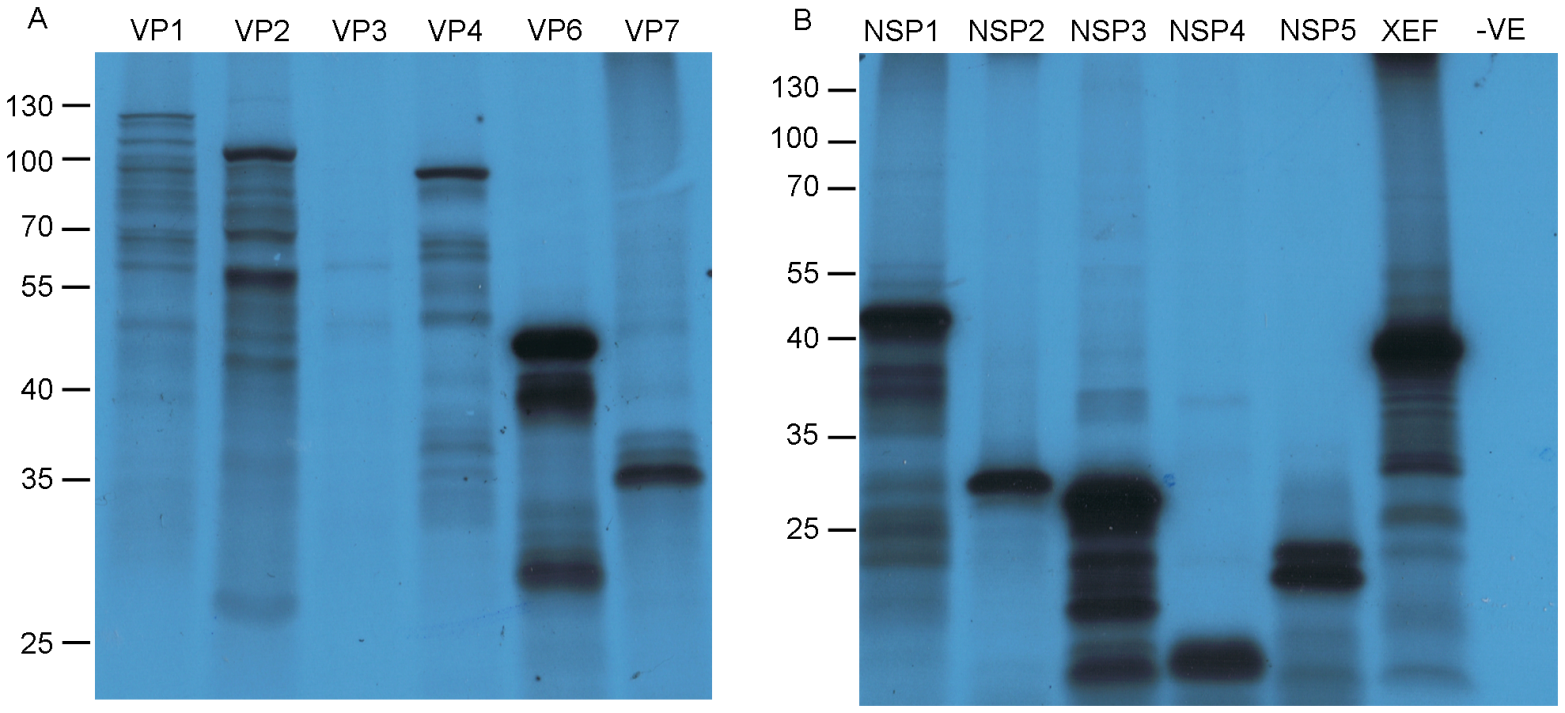
*In vitro* translation of RV proteins using S1 - 11 *in vitro* transcribed ssRNAs. *In vitro* translation using all *in vitro* transcribed RV ssRNAs as templates for protein synthesis. 500 ng of ssRNA was incubated in RRL with ^35^S L-methionine for 3 hours and analysed using SDS-PAGE. The dried gel was exposed to X-ray film for 36 hours. Panel A, 15% SDS - PAGE, contains the *in vitro* translated RV structural proteins; VP1, VP2, VP3, VP4, VP6 and VP7, respectively. Panel B, 12% SDS-PAGE, contains the *in vitro* translated RV non-structural proteins; NSP1, NSP2, NSP3, NSP4 and NSP5, respectively. Lane 6: Xenopus elongationfactor 1α (XEF) (positive control); lane 7: no ssRNA, (negative control). The sizes of protein markers run alongside during SDS-PAGE are indicated in kDa to the left of the autoradiographs.

There are many published examples of *in vitro* translation of RV RNAs, derived from either DLPs or transcribed *in vitro* from cDNA [39], [40], [41], [42]. To ascertain whether the *in vitro* transcribed RV ssRNAs function as mRNAs, we tested them *in vitro* using rabbit reticulocyte lysates (RRL). The efficiency of translation was higher with ssRNA transcripts which had been post-transcriptionally capped (unpublished data). We performed individual *in vitro* translations with all 11 post-capped RV RNAs transcribed *in vitro* from cDNA**.** Figure 4 shows an autoradiograph of the translation products. All 11 RV RNAs generated proteins of the expected size, with the largest proteins encoded by segments 1–4 being synthesised at lower efficiencies. Higher expression of the smaller RV proteins, even at equimolar amounts of ssRNA, has been observed before [43]. Figure 4, panel A shows successful synthesis of the RV structural proteins. Segment 3 RNA, VP3, has a significantly lower level of protein expression when compared to segment 2 RNA, VP2, which encodes a protein of comparable size. The discrepancy between the protein yields could be due in part to the fact that *in vitro* transcription from the RF segment 3 template produced two products (Figure 1, lane 3) only one of which corresponds to the full length RNA. Alternatively the presence of at least two predicted stable stem structures in RNA3 may have contributed [32]. The level of VP6 expression is very high (Fig. 4, panel A, lane 5). This is not an artifact of a high methionine residue content relative to the protein’s molecular size when compared to the other RV proteins. It has been hypothesised that VP6 may have its own segment-specific enhancer sequence promoting its own translation [44], and a stable stem loop structure has been mapped using *in silico* analysis to the same region [32]. Figure 4, panel B shows the synthesis of the RV non-structural proteins. In panel B, lane 4, NSP4 migrates faster than would be expected based on its molecular weight of 20 kDa, highlighted by comparison with the migration of NSP5 (21 kDa) (lane 5). This phenomenon has been observed before [42], [43]. The molecular sizes of NSP4 and NSP5 are very similar, and both proteins are calculated to possess a small negative charge at pH 8.8 (Scripps web protein calculator v3.3). It is possible that the protein product seen is actually a truncated version of NSP4 after cleavage by proteases in the RRL. N- and C-terminal protein sequencing would identify a truncation if this was the case. The translation reactions of all RV RNAs show several bands migrating faster than the molecular weight of the authentic protein encoded in the ssRNA template. This is likely due to premature termination of protein synthesis, and Western blot could be used to reconfirm the identity of the largest protein in each reaction., The multiple band profiles are not RV specific as they are observed in both the RV samples and the XEF positive control (Figure 4, panel B). As the largest product from each reaction migrates at the expected size of the RV encoded in the ssRNA, we concluded that all RF RV ssRNAs can function as templates for protein synthesis using the mammalian translational machinery.

**Figure 5:**
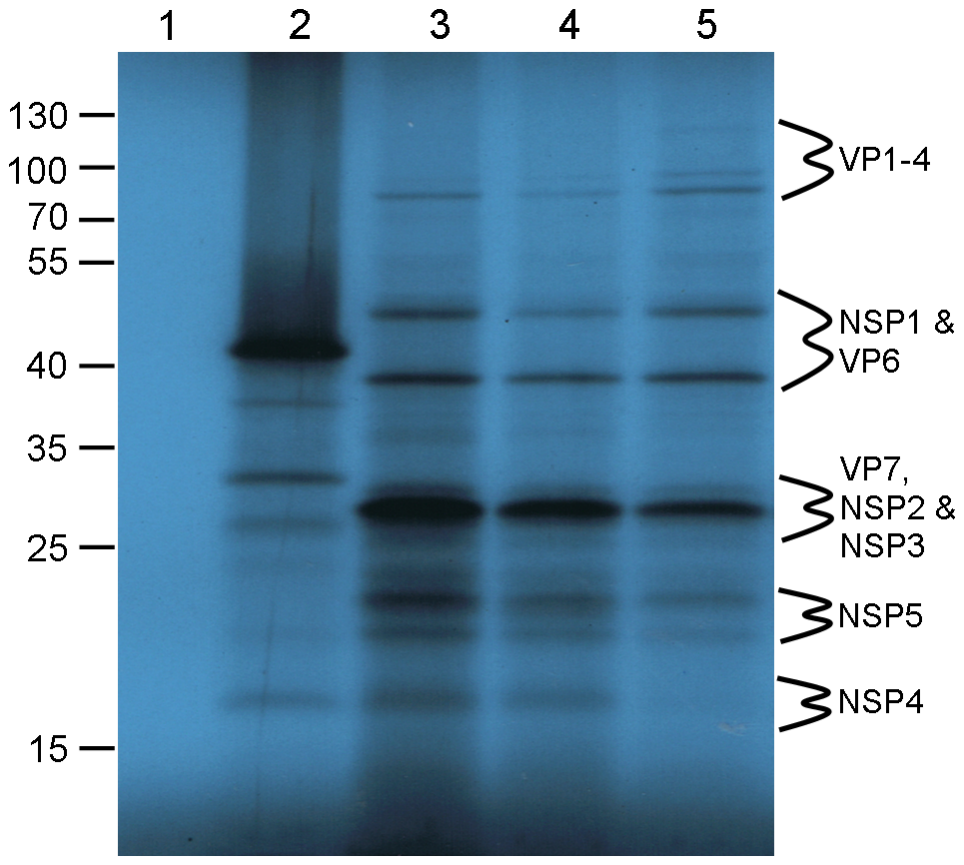
In vitro translation of RV proteins from cohorts of cDNA-derived and DLP-derived ssRNAs. *In vitro* translation of RV DLP derived ssRNAs. 1 µg of capped DLP ssRNA was incubated in a RRL as described, electrophoresed on a 15% SDS-PAGE alongside PageRuler™ protein markers (in kDa) and exposed to X-ray film for 4 days. Lane 1: no ssRNA; lane 2: XEF ssRNA; lanes 3 & 4: S1–11 post-capped or co-capped ssRNA respectively; lane 5: RV ssRNAs from RV RF strain DLPs. The sizes of protein markers run alongside SDS-PAGE are indicated in kDa to the right hand side and were used to predict the location of RV proteins.

We also tested the translation capacity of the DLP-derived ssRNAs of the strains we had used. We incubated DLP-derived transcripts and mixtures of all 11 segments from co-capped or post-capped ssRNAs of the RF strain, in RRL. Figure 5, lanes 3 and 4 demonstrate that cohorts of the post-capped ssRNAs are translated with greater efficiency than cohorts of co-capped RNAs. Comparison between lanes 3, 4 and 5 reveals highly similar protein translation profiles for the cDNA-derived and DLP-derived ssRNAs of the same strain (RF). While the presence of every RV protein would require complex Western blot analysis, the striking resemblance between the protein profiles of synthetically generated and DLP-derived ssRNA cohorts provides compelling evidence that the ssRNAs are functional templates for protein synthesis.

The poor translation of segment 3 RNA of the RV RF strain is supported by similar findings with the bovine RV UK Compton strain [43] whose sequence is very similar to that of the RF strain. We investigated the transcription products of segment 3 cDNA of the SA11 simian RV strain which encodes a similar VP3 (cloned into a T7 Pol transcription cassette from genomic dsRNA by RT-PCR) and obtained only a single RNA band of the correct size (Figure S4, panel A, VP3). To ascertain whether the SA11 ssRNA is a more efficient template for protein synthesis, we compared segment 3 *in vitro* transcripts of RF and SA11 RV strains as templates of protein synthesis *in vitro*. The RNAs of both strains produced very similar protein expression profiles, but the amount of protein translated from the RNA of SA11 is at least double that of RF (Figure S4, panel B, lanes 2 & 3).

### Optimisation of ssRNA Transfection using an eGFP Reporter mRNA

**Figure 6:**
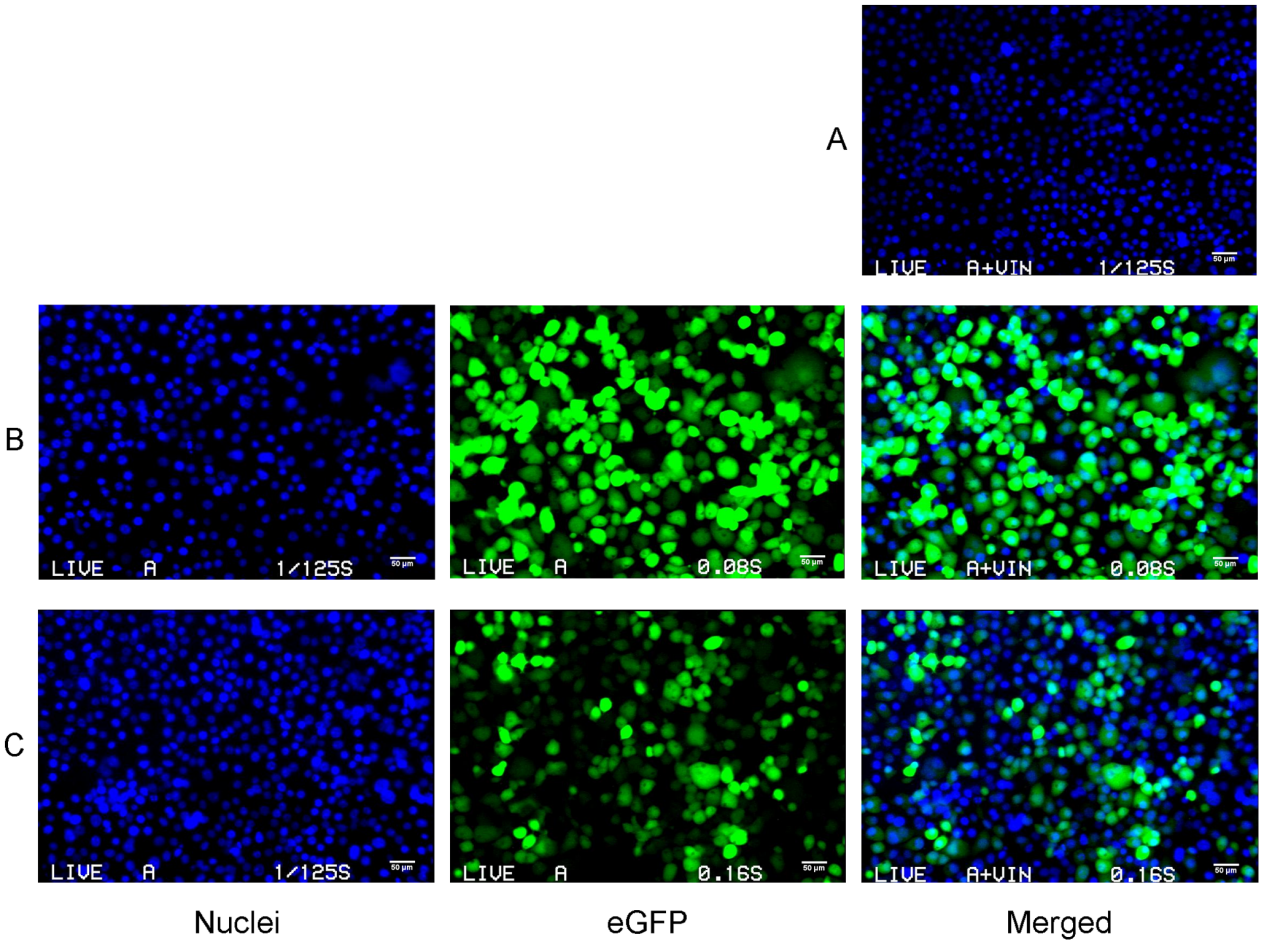
Comparison of fluorescence between transfected eGFP RNA species. COS-7 cells at 80% confluence, incubated in Opti-MEM I media were transfected with eGFP ssRNA using Lipofectamine 2000. Cells were stained with Hoechst 33342 and live imaged in an epifluorescence microscope at 24 hours post transfection. Panel A: mock transfected cells. Panels B & C: cells were transfected with 750 ng of eGFP ssRNA either post-capped or co-capped, respectively. Cells were exposed to UV (nuclei staining), blue light (eGFP excitation) or merged, respectively.

To optimise transfection with large ssRNAs, we initiated pilot experiments using a specifically constructed reporter eGFP mRNA. We transfected COS-7 cells in parallel with *in vitro* synthesized ssRNA and a DNA plasmid (pEGFP-N1) both of which encode an identical eGFP ORF (718 bp) under the control of the same Kozak enhancer sequence. The ssRNA was either co-transcriptionally or post-transcriptionally capped and then polyadenylated. Figure 6 demonstrates that the post-capped ssRNA was 23% more efficient as a template for protein synthesis than the co-capped ssRNA (quantitation not shown). The expression of eGFP was detectable with as low as 5–10 ng of transfected ssRNA, however, the transfection efficiency in MA104 cells was lower than that seen in COS-7 cells (unpublished data). A comparison of different transfection reagents (Lipofectamine 2000 (Invitrogen), Jet prime (PolyPlus), X-tremeGENE (Roche), turbofect (Fermentas) and TransIT-mRNA (Mirus) demonstrated that for ssRNA transfection the Mirus reagent was optimal, whereas for cDNA transfection it was Lipofectamine 2000 (unpublished data). We used a mammalian (rather than a RV) based expression cassette for the eGFP control ssRNA as previous data have shown that reporter genes (encoded by DNA or RNA) flanked with RV UTRs from gene 6 are not efficiently translated [37], [38]. Thus an eGFP ORF flanked with viral UTRs might be equally poorly translated and provide an inadequate positive control. Additionally the presence of the RV UTRs would not answer the question as to whether the UTRs were solely responsible for the efficient translation. The use of the eGFP reporter mRNA was crucial to validate the transfection experiments.

### Transfection of *in vitro* Transcribed and DLP-derived RV ssRNAs

**Figure 7:**
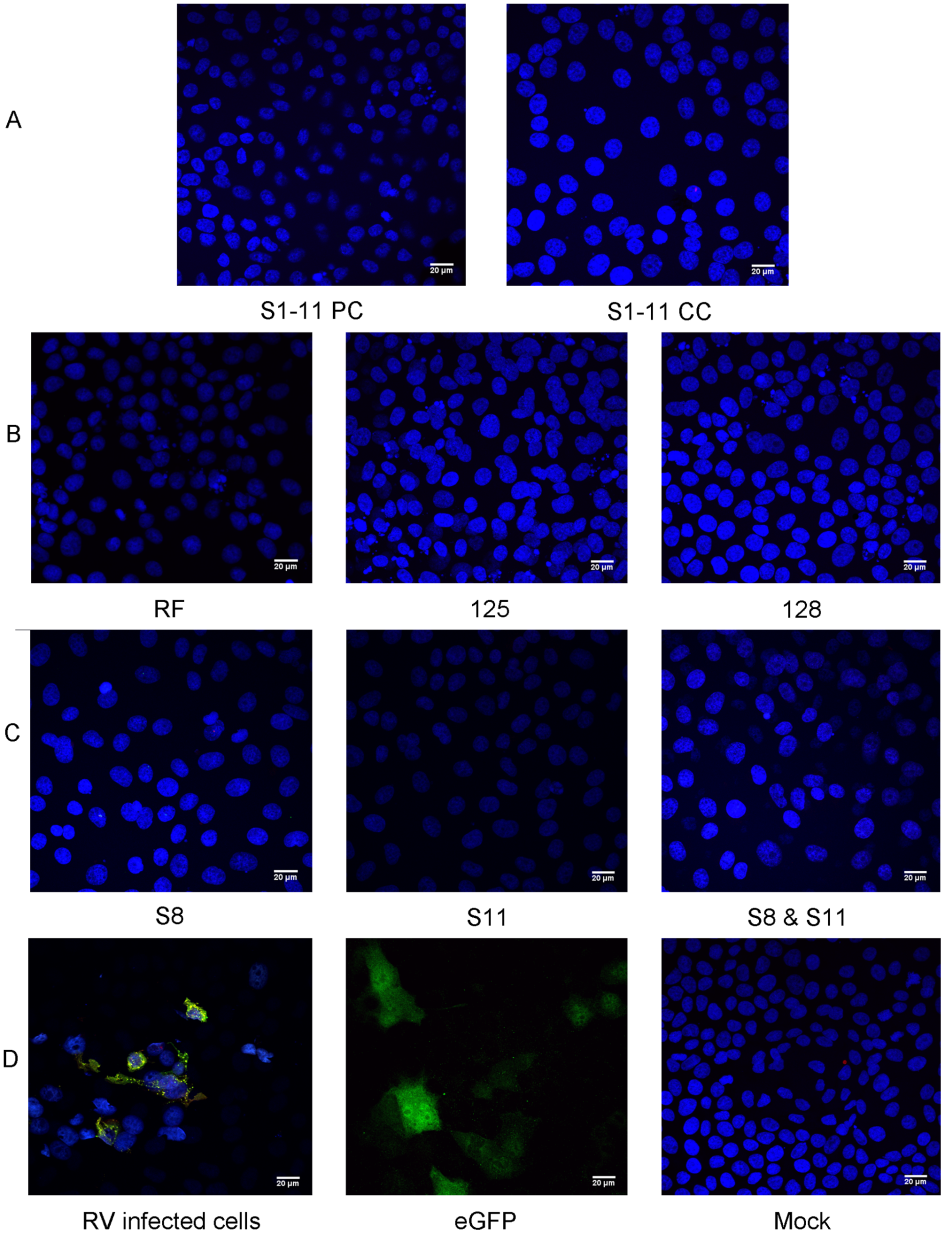
Transfection of COS-7 cells with *in vitro* transcribed ssRNAs encoding RV ssRNAs and DLP derived ssRNAs. COS-7 cells at 80% confluence were transfected with ssRNAs encoding RV proteins using Mirus transfection reagent. Cells were fixed at 24 hours post transfection and stained with NSP2 and NSP5-specific antibodies (Table S4). Images were analysed by confocal microscopy. Panel A: transfection with cohorts of *in vitro* transcribed ssRNAs, 1 µg of S1– S11, post-capped (PC) or co-capped (CC) respectively, stained for both NSP2 and NSP5. Panel B: 1 µg of DLP derived ssRNAs from RV strains RF, 125 and 128 respectively, stained for both NSP2 and NSP5. Panel C: individual or co-transfection of 500 ng of ssRNAs S8, S11 or both stained for NSP2, NSP5 or both, respectively. Panel D: immunofluorescence of control MA104 cells infected with RF RV, stained for NSP2 and NSP5, transfection control with 1 µg of eGFP ssRNAs (autofluorescence), mock transfection stained for NSP2 and NSP5. Cell nuclei were stained with Hoechst 33342 in all panels. Scale bars: 20 µm.

We transfected COS-7, MA104 and 293T cells with cohorts of the *in vitro* transcribed ssRNAs in Opti-MEM I, using the Mirus transfection reagent. Cells were transfected with cohorts of ssRNAs, ranging in mass from 10 ng –2 µg (each segment representing 1/11^th^ of the total mass) or equimolar concentrations of each ssRNA. Protein expression or nascent viral formation was sought. All three cells lines showed significant cytopathic effects after 24 hours, progressing to almost total cell destruction (60 - 80%) at day 3 post transfection. Media or frozen and thawed lysates from transfected cells were inoculated onto fresh MA104 monolayers to test for infectious virus but none was detected at 7 days. It was recently shown that BTV rescue was enhanced by double transfection of ssRNAs [45]; we thus transfected two cohorts (S1–S11) of RV ssRNA segments 16 hours apart. We hypothesised that, S1, S2, S3, S6, S8 and S11, encoding VP1, VP2, VP3, VP6, NSP2 and NSP5, respectively, might form the minimum essential group of proteins for initial viroplasm and DLP formation. The second transfection step contained all 11 ssRNAs. (We also added the SA11 RNA 3 to the RNAs of the RF RV strain in some experiments). Viable viral progeny was not detected (in MA104 cells) from the cell extracts of these experiments. Transfections (single or double) of cells with DLP-derived ssRNAs of five RV strains (RF, SA11, OSU, 125 and 128) were similarly unsuccessful (unpublished data). To ascertain whether RV ssRNA transcripts had actually entered cells, the cohort of S1–S11 ssRNAs was mixed with eGFP ssRNA and transfected as an ensemble of 12 ssRNA transcripts. eGFP expression was observed in many cells, however the supernatant from this experiment yielded no progeny virus (unpublished data). We hypothesised that the full length RV ssRNAs were not autonomously infectious, but it was unclear whether this was due to a lack of protein translation, potent innate immune responses, a high degree of cytotoxicity exerted by the transfected RNAs or a combination of more than one of these factors. To investigate this we transfected cells and sought RV protein expression using specific antibodies. Figure 7 shows the results for individual and ensemble ssRNA transfection experiments in COS-7 cells. Cells were transfected with varying amounts of a variety of ssRNAs, either cDNA- or DLP-derived, and incubated for 24 hours prior to fixing and staining with RV specific antibodies (for details see legend of Figure 7). There was no detectable NSP2 or NSP5 expression in any of the transfection experiments (and consequently no VLS formation). Similar observations were made in MA104 and 293T cells, VP1 expression was similarly undetectable (unpublished data). We then hypothesised that expressing RV proteins in cells prior to RV RNA transfection might be necessary to permit RV protein synthesis from ssRNAs under these conditions. Co-expression in *trans* of NSP2 and NSP5 six hours before, or simultaneously with, RV ssRNA transfection failed to yield infectious progeny virus (unpublished data). An MA104 cell line constitutively expressing NSP5-eGFP [46], was transfected with S8 ssRNA encoding NSP2, but we saw no VLS formation by immunofluorescence (unpublished data).

### Transfection with Polyadenylated RV ssRNAs

It was plausible that rapid delivery to cells of highly structured ssRNAs in large amounts might trigger the innate immune response, possibly leading to cell cycle arrest through mechanisms such as interferon induction. As RV ssRNAs are not naturally polyadenylated whereas the reporter eGFP RNA was, we hypothesized that this might selectively flag the viral RNAs for degradation prior to translation [47]. We therefore polyadenylated the RV ssRNAs post-transcriptionally. We were aware that the disruption of the native 3′ end, which is highly conserved in RVs, could have significant negative implications for replication and packaging of ssRNAs [37], [48], [49], [50], [51]. However, had protein expression occurred this might have facilitated authentic ssRNA translation from a subsequent transfection. We performed this experiment with individual *in vitro* transcribed RV ssRNAs and a variety of ssRNA ensemble mixtures. Figure S5 shows the successful polyadenylation of 4 ssRNAs (S1, S8, S9 & S11). However, this did not lead to detectable gene expression after transfection (Figure S6). Analogous experiments with polyadenylated DLP-derived ssRNAs did not lead to detectable protein expression either and transfection of the polyadenylated RV ssRNA into Vero cells, which are deficient in interferon production [52], [53] also did not lead to detectable RV protein expression (unpublished data).

### RV Protein Detection by Western Blot

**Figure 8:**
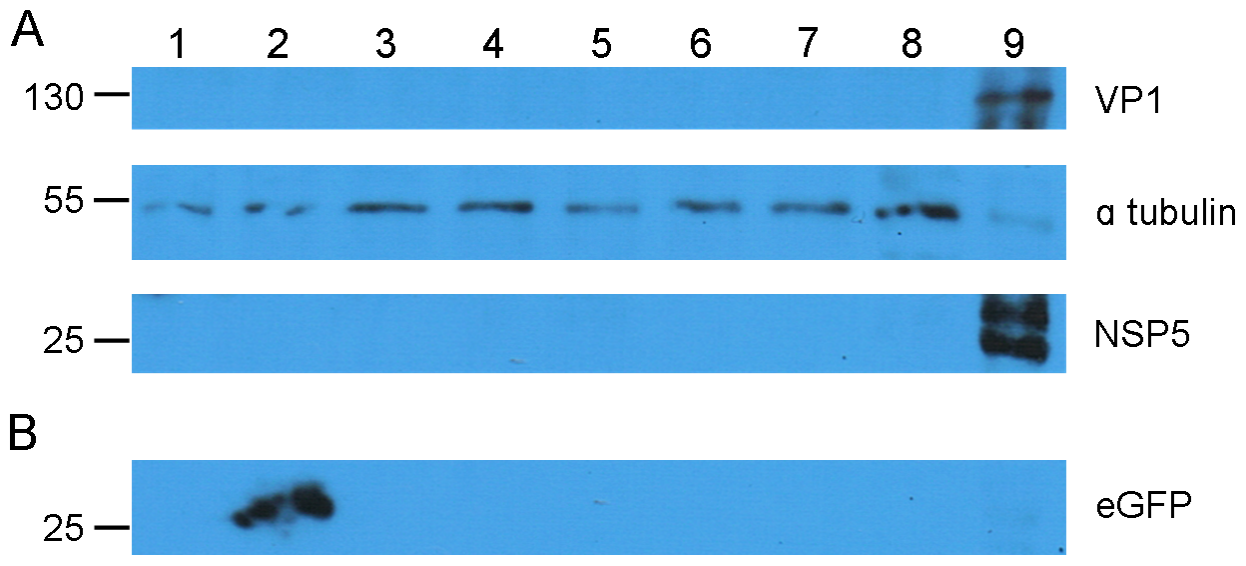
Absence of VP1 and NSP5 expression from transfected ssRNAs by Western blotting. COS-7 cells were transfected with 1 µg of RV ssRNA using the Mirus TransIT™ mRNA transfection reagent. Expression of RV proteins from cell lysates was sought by Western blot. Panel A, the membrane was split into three sections, to ascertain the presence of VP1, 170–70 kDa, loading control α tubulin, 70 - 40 kDa and NSP5, 40–15 kDa. Each section was incubated with the respective primary and secondary horseradish peroxidase (HRP) conjugated antibody (Table S5). Panel B, corresponding to the 40–15 KDa portion of membrane A which was reprobed for eGFP (Table S5). Proteins were visualised using the ECL Western blot detection reagents, light sensitive film was exposed to membranes for varying lengths of time depending on band intensity. Lane 1: mock; lanes 2–8 *in vitro* transcribed or DLP derived ssRNAs of: 2: eGFP, 3: S1– s11, 4: S1, 5: S11, 6: DLP RF, 7: S1 polyadenylated, 8: S11 polyadenylated; 9: COS-7 infected RV RF cell lysate.

We sought evidence of RV protein expression by Western blotting COS-7 cell lysates transfected with RV ssRNAs. Figure 8, panel A clearly shows that neither VP1 nor NSP5 proteins were detected from cells transfected with DLP-derived or cDNA-derived *in vitro* transcribed RV ssRNA, with or without polyadenylation. To validate the Western blot procedure, the membrane section was reprobed using an eGFP-specific antibody (Figure 8, panel B, lane 2); eGFP protein was easily detected. To address the question of differing viral protein translation efficiencies, we also examined the expression of two additional structural proteins, VP2 and VP6 (Figures S7 and S8). Our selection of these two proteins was due to the high copy number required for RV particle formation, VP6 is known to be the most abundantly expressed structural protein [54] and has a segment specific enhancer in addition to the RV specific 3′ translational recognition sequence [55], [56]. We concluded that neither unmodified nor polyadenylated RV ssRNAs, obtained from either *in vitro* transcribed cDNAs or from DLPs, can act as efficient templates for protein synthesis when transfected into cells and therefore, by definition, were not autonomously infectious.

### Recombinant Fowlpox virus Expressing T7 RNA Polymerase as a Tool to Drive Intracellular Transcription of PCR Synthesised RV Segmental Amplicons

A recently developed RG system for a positive sense ssRNA virus, the enteropathogenic norovirus, utilised recombinant fowlpox virus (FPV) which expresses T7 RNA polymerase (FPV-T7) [29], [57]. FPV cannot replicate in mammalian cells [58] and is of very low cytopathicity [59]. Published data on the helper virus-dependent RV reverse genetics system has shown RV and vaccinia recombinant virus superinfections in combination with transfected DNA templates can produce recombinant RVs [23], [25]. To test this system, we used infection with FPV-T7 to transcribe RV ssRNA from transfected linear cDNAs intracellularly (the amplicons are shown in Figure S2).

**Figure 9:**
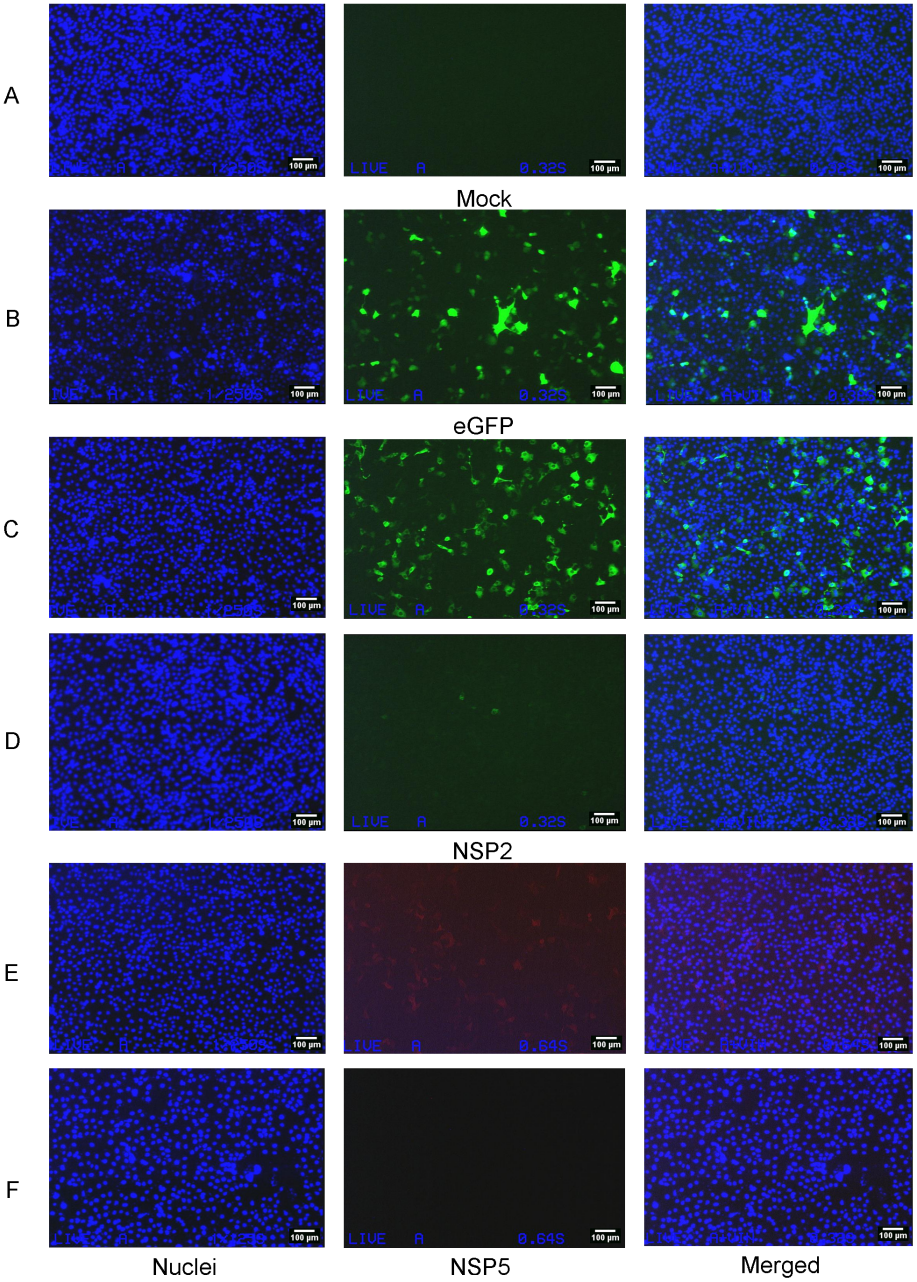
FPV-T7-driven intracellular transcription and translation from transfected linear DNA templates of NSP2 and NSP5. COS-7 cells infected with FPV-T7 Pol for 1 hour and transfected with 1 µg of linear PCR amplicon encoding either RV segments 8 or 11 or EGFP. pcDNA3-NSP2 and pT7V-NSP5 were used as protein controls. Cells were incubated for 24 hours, prior to fixing and staining for RV proteins as previously described. Cells transfected with EGFP were only stained with Hoechst 33342. Samples were imaged using a fluorescence microscope. All panels were exposed to UV light, panels A, B, C, and D were exposed to blue and panels E and F were exposed to green wavelengths of light. Panel A: mock transfection; panel B: transfection with T7EGFP; panel C: transfection with pcDNA3-NSP2; panel D: transfection with T7RFS8; panel E: transfection with pT7V-NSP5; panel F: transfection with T7RFS11. Cell nuclei were stained with Hoechst 33342. Scale bars: 100 µm.

Once we had validated the experimental protocol with the T7-eGFP amplicon, we examined whether intracellular transcription of RV positive sense RNAs from transfected cDNA could enhance protein expression, or produce infectious RV particles. We transfected cDNA templates of segments 11 (T7RF11) and segment 8 (T7RFS8) into COS-7 cells. Figure 9 shows the absence of detectable protein expression when linear DNA templates encoding either NSP2 (panel D) or NSP5 (panel F) were transfected. Several cells (<20 in total) in panel D (transfected with S8 [NSP2] cDNA) showed green fluorescence. However, this was also seen in the FPV-T7-only infected control (Panel A). We attributed this effect to non-specific binding of NSP2 antibody to apoptotic cells. These artifacts were not observed with the NSP5 specific antibody (panel F). When the cohort of 11 RV cDNAs was transfected into FPV-T7 infected cells, no infectious RV progeny was rescued (unpublished data). It was reasonable to assume that the cDNA amplicons were transfected successfully into cells and probably acted as templates for intracellular transcription, as eGFP was expressed from a transfected linear amplicon however the RV ssRNA transcripts were apparently not competent for efficient translation.

### Utilising VLS to Rescue RV after ssRNA Transfection

**Figure 10:**
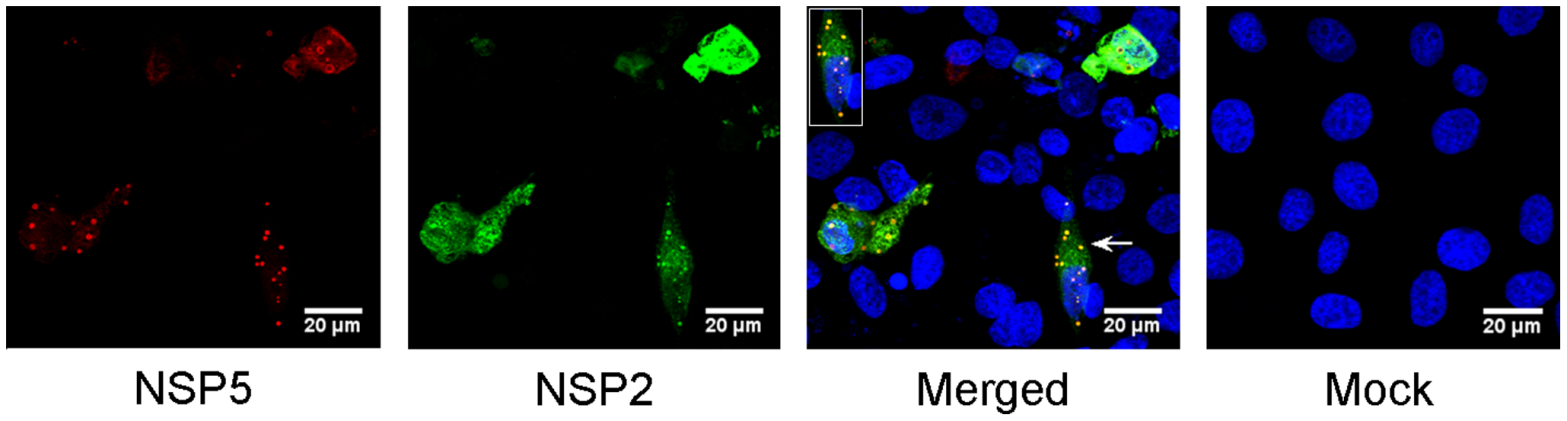
Viroplasm-like structure (VLS) formation. MA104 cells were infected with FPV-T7 for one hour before co-transfection with pT7V-NSP2 and pT7V-NSP5 plasmids using Lipofectamine 2000. Cells were fixed at 24 hours post transfection and stained for NSP2 and NSP5. Cell nuclei were stained with Hoechst 33342. VLS and mock panels are merged images of UV and the other two composite excitations for NSP2 or NSP5. Images were visualised by confocal microscopy. Arrows indicate VLS and location of enlarged inset images. Scale bars: 20 µm.

We reasoned that the creation of pre-existing VLS from co-expression of plasmids encoding NSP2 and NSP5, under the control of T7 Pol promoter, might create a cellular environment more favourable to RV protein translation from ssRNAs. We tested this hypothesis in MA104 cells where VLS were formed (Figure 10). Subsequent transfection with the cohort of ssRNAs or just segment 1 alone did not yield protein expression or rescue infectious virus. VLS were also formed in COS-7 and Caco-2 cells but upon RV ssRNA transfection we did not observe corresponding RV protein expression or rescue of infectious virus (unpublished data).

## Discussion

Exploring RV replication at the molecular level has been hampered by the lack of a technology which permits rescue of genetically defined RVs in the absence of a helper virus. More recently several helper virus-dependent RG systems for RVs have been established, but they all require a strong selective pressure against the helper virus, are cumbersome and are not universally applicable to all RVs or all RV segments [22], [23], [24], [25]. Furthermore the possible effects of strong selection methods (antibodies or siRNAs) on the formation of recombinant RVs are unknown. For several viruses of other genera of the *Reoviridae* family ssRNAs of positive sense polarity produced by methods analogous to those investigated in this paper (*in vitro* from cDNAs or from viral cores/subviral particles) can, under the correct conditions, initiate a replication cycle, permitting rescue of infectious virus progeny [18], [19], [20], [27], [45]. The approach of using RV ssRNA to develop a helper virus-free RG system was supported by the finding that RV replication profoundly suppresses certain host cell processes [38]. Transfection of RV DLPs into susceptible cells can initiate an infection [60], yet a protocol to engineer a helper virus-free RG system for RVs has not as yet been published.

We modelled our approach on the successful protocol for engineering a RG system in viruses of the *Orbivirus* genus [20], [21], [27]. Since both BTV and RVs share many structural and replication characteristics, we used the FLAC procedure [30] to amplify and subsequently clone the RV genome, a technique which has recently been applied to the amplification of cDNAs of other multisegmented dsRNA viruses [31].

Shotgun cloning RV cDNAs necessitated complete sequencing of several clones for each segment. The sequences obtained from the clone library were nearly identical to those already available in GenBank which have been used in a variety of studies [61], [62], [63], [64], [65], [66]. We assumed that the GenBank sequences of the RV RF strain were an accurate representation of their genetic composition and the majority of mutations detected occurred in the UTRs of each RNA, thus not affecting the protein structure. It is possible that the mutations could affect packaging efficiency but it is generally accepted that the UTRs and part of the adjacent ORFs of RV RNAs are likely to be important for efficient packaging [45], [67] and the importance of viral protein-RNA and protein-protein functions was not related to the major obstacle we discovered; inefficient translation from full length RV RNAs either directly transfected into cells or synthesised from cDNAs transfected into the cytoplasm. Using the clonal sequences, consensus sequences were generated from which we constructed 11 RV T7 Pol transcription templates which permitted *in vitro* synthesis of positive sense RNAs with native 5′ and 3′ ends for each of the 11 RV genome segments. Synthesis of a full genomic complement of RV RNAs using this method has not previously been reported, and the system may have applications in other aspects of RV replication such as RNA analysis. We also synthesized DLP-derived ssRNAs from CsCl gradient purified DLPs [35], [36].

We noted double bands in the *in vitro* ssRNA transcription products of RV segments 3 and 6. One band of each was consistent with the full length product; the other one was smaller. Another publication has found comparable results when *in vitro* transcribing the influenza H5N1 virus HA RNAs [33], where faster migrating bands were attributed to premature *in vitro* transcription termination due to a type II RNA hairpin loop terminator [33]. This is a plausible assumption for RV segments 3 and 6 as we know that RV ssRNA segments are highly structured [32]. When we produced a positive sense ssRNA transcript of a segment 3 cDNA of the simian SA11 rotavirus strain only one product of the expected length was obtained.

We demonstrated successful translation of all 11 RV proteins derived from both the *in vitro* transcribed and the DLP-derived ssRNAs in the RRL. When cohorts of all 11 ssRNAs obtained from DLPs or cDNAs were used they translated into identical protein patterns, suggesting that the cDNA-derived ssRNAs were as functionally capable as DLP-derived RNAs for the synthesis of RV proteins.

Cohorts of DLP-derived or *in vitro* transcribed ssRNA molecules were co-transfected into a variety of cell lines capable of supporting RV infection (COS-7, MA104, Vero, 293T cells), but were not infectious as judged by the failure to rescue infectious virus from transfected cell extracts by subsequent passages on MA104 cells. The transfection procedure for RV ssRNAs, similar to that of other orthoreoviruses [20], [21], was validated using a reporter mRNA encoding eGFP.

A large variety of strategies were assessed to attempt to generate a functional RG system. The recently published approach for AHSV RG indicated an improved yield of rescued virus using a dual transfection system where two different cohorts of ssRNAs were transfected 16 hours apart [21]. We tested this using two different cohorts of RV ssRNAs (1. S1, S2, S3, S6, S8 & S11; 2. S1–S11; transfected 16 h apart) but without success.

Cytotoxicity following ssRNA transfection was observed in all cell lines used. The level of cytotoxicity did not correlate with the total amount of RNA transfected as identical masses of transfected eGFP ssRNA resulted in protein expression and comparable experiments with RV ssRNAs did not yield protein expression. Cytotoxicity was also observed when cohorts of 6 ssRNAs or individual RNAs at varying masses were transfected, and in no case was protein expression detected. Conditions for ssRNA transfection were extensively and carefully explored and particular attention was paid to replicate the published experimental conditions for large cohorts of ssRNAs [20], [27], [45], [68], [69].

To examine protein expression efficiency, we tested protein expression from transfected ssRNAs using immunofluorescence and Western blotting techniques. We investigated VP1, VP2, VP6 NSP2 and NSP5 expression from their encoding RNAs in a variety of transfection experiments, but we were unable to detect RV protein expression at any time. Even transfected DLP-derived ssRNAs were incapable of intra-cytoplasmic protein synthesis. Transfected naked RV ssRNAs, unlike the co-transfected eGFP ssRNA, were not competent templates for protein synthesis. It is worth noting that both BTV and AHSV ssRNAs synthesised from cores, which also lack a 3′ polyadenylation sequence, can form nascent viruses post transfection into BSR cells [21], [27] but this phenomenon is yet to be described for RV. From these data we concluded that despite the ssRNAs being used for *in vitro* translation in RRL, efficient translation inside mammalian cells was blocked.

The lack of success with the procedures discussed above led us to question whether the transfected ssRNAs were functional as templates for protein synthesis once present in cells as the RNAs produced were clearly recognised by mammalian translational machinery *in vitro*. We dismissed the possibility that the ssRNAs were rapidly being degraded because there is strong published evidence showing that RV ssRNAs are stable post transfection into RV infected or uninfected cells [70]. In addition our experiments using the control eGFP encoding ssRNA demonstrated that RNA stability and translation of mammalian transcripts were not an issue. Attempts at camouflaging RV ssRNAs to appear more akin to a cellular transcript were also unsuccessful in enhancing protein expression. Furthermore, protein expression was not detected when ssRNA transcripts synthesised from purified DLPs were transfected into cells yet the same DLP preparations were capable of initiating an infection after transfection. Our findings clearly indicate when viral transcriptional machinery is used to synthesise RV ssRNAs transcripts, the intracellular location of transcription is crucial. Therefore we concluded that our RV ssRNAs must not have been recognised efficiently after transfection.

It is possible that nascent RV proteins or particles were formed during our experiments but at a level that was undetectable. We attempted to address these issues by repeating transfections of ssRNAs to enhance viral protein translation which has been shown to enhance viral yields [21], [45], analysed concentrated amounts of cell lysates (20/30% of cell lysate per lane) transfected with ssRNAs by Western blot for a variety of RV proteins. Media and lysates were used to inoculate fresh MA104 monolayers and incubated for 7 days in the presence of trypsin to activate potential nascent RV. We concluded, based upon the cumulative data, that the problem was most likely due to a block in protein translation rather than to inadequate detection systems because after 7 days even very low titers of nascent viruses would show RV viral protein expression.

The paradox between successful expression of RV proteins in RRL and undetectable expression in permissive cells from transfected ssRNAs could be due to an increased concentration of eIF-4E in RRL [71]. Over expression of eIF-4E has been shown to increase synthesis of mRNAs with highly structured 5′ ends [72], it is therefore plausible that intact DLPs are able to recruit this initiation cofactor whereas ssRNA alone cannot and only in RRLs which possess increased eIF-4E concentrations can protein expression be achieved to detectable levels. This would also account for the observation that transfected DLPs are infectious yet DLP-derived *in vitro* transcribed ssRNAs are not.

In order to test the potential of a RV helper-virus free system based on cDNA transfection we used a FPV-T7 Pol recombinant virus [57]. We hypothesized that a transfection system based on cDNA, followed by intracellular transcription might facilitate translation of RV proteins and rescue of infectious virus progeny. The eGFP amplicon as a reporter linear cDNA molecule was successfully transcribed and translated by this method, but again we observed a lack of RV protein translation and virus recovery when FPV-T7 recombinant-infected cells were transfected together with 11 RV cDNAs. Even NSP2 and NSP5 provided *in trans* (leading to the formation of VLS) [73] did not enable RV protein expression or viral rescue. The delivery of nascent RV ssRNAs into the cytoplasm from inside the cell was not sufficient to enhance protein translation. Based on these data we concluded that full length RV ssRNAs are non-infectious and incapable of efficient translation in the cell lines investigated, whether derived from cDNA- or ssRNA-based transfection.

The observation that both, DLP-derived ssRNAs and *in vitro* transcribed ssRNAs from cDNAs, were not infectious when transfected into susceptible cells was puzzling particularly since DLPs transfected into cells can initiate protein expression and infection [60]. Yet ssRNAs produced from these same particles *in vitro* were not capable of acting as templates for translation when transfected into permissive mammalian cells. Clearly there must be something crucial and as yet unexplained about the cellular location or the way in which the ssRNAs exit the DLPs in the cytoplasm which permits viral protein synthesis and the subsequent steps of RV replication. Possibly ssRNAs exiting DLPs interact immediately with ribosomes.

RV mRNAs are not polyadenylated. NSP3 binds the 3′ consensus translation enhancer sequence, UGACC [41], but also the carboxy terminal domain of eIF4GI, a functional homologue of eIF4G, in the same region as PABP but with a higher affinity [74], [75]. Thus, NSP3 has been implicated in circularising RV ssRNAs, facilitating greater expression of the proteins from non-polyadenylated ssRNA [75]. Conversely, it has been suggested that NSP3 is not required for protein expression [76], [77], [78]. NSP3 has also been shown to shutdown host cell protein synthesis by displacing PABP on cellular mRNAs [79]. It is known that host cell protein synthesis is shut down almost completely during RV infection through eIF2α phosphorylation [80]. Perhaps the key to facilitating RV recovery from ssRNA lies in achieving a significant shutdown of host cell protein synthesis by retention of polyadenylated cellular mRNA from the nucleus [38]. Our data show that masking the RV ssRNAs by polyadenylating them so that they appeared mRNA-like in structure did not lead to enhanced translation or virus recovery; it is likely that the stability of these polyadenylated ssRNAs would be equivalent to that of normal mRNAs.

The sudden appearance of large amounts of highly structured RV ssRNA molecules [32] in cells could trigger the innate immune response, through the RIG-I and STAT pathways [81]. The innate immune response can be blocked in cells which constitutively express proteins inhibiting these pathways [81], and such cell lines may be useful in future exploration of a RV RG system. The continued pursuit of a ssRNA-based, helper virus-free system is valid because of the report that polyadenylated reporter ssRNAs are more efficient templates for translation when transfected directly into the cytoplasm then when transcribed in the nucleus from transfected cDNA [38].

The recent *in vitro* reconstitution of BTV using *in vitro* translation and ssRNA templates may be an approach worthy of exploration to examine RV packaging signals and to construct recombinant RV particles [82]. As the system does not depend on viral particle formation in cells, cellular factors such as innate immune responses are not a consideration. Obviating the need to provide proteins in *trans,* this system may be useful for generating RVs with mutations which are lethal or non-viable in cell-dependent systems. As all the RV proteins can be translated from ssRNAs *in vitro,* and based on the knowledge that VP2 and VP6 form DLP-like particles, this approach maybe a promising direction for further research into RV RG systems.

## Materials and Methods

### Cells

The majority of experiments were performed with MA104 cells (ATCC CRL-2378.1). Additional experiments were in COS-7 cells (ATCC CRL-1651), Vero cells (ATCC CRL-1586) and 293T cells (ATCC CRL-11268). The cells were maintained in Dulbecco’s Modified Eagle Medium (DMEM, PAA; Invitrogen) which was supplemented with non-essential amino acids (0.1 mM), penicillin (100 U/ml), streptomycin and (10 µg/ml) and 5% (for Vero cells 10%) fetal calf serum (FCS). An MA104 cell line stably expressing a C-terminal fusion of NSP5 to eGFP (termed NSP5-eGFP) [46]; donation from Dr Oscar Burrone, Trieste) was cultured as described above. All cells were cultured at 37°C in 5% CO_2_/air atmosphere in a Sanyo incubator.

### Viruses

The following RV strains were used: bovine RF (G6P6 [1]), simian SA11 (G3P [2]), porcine OSU (G5P [7]) and human bovine reassortants 125 and 128 containing gene rearrangements in segments 8 and 11 (G6P [6]) [83]. Rotaviruses were propagated and their infectivity (in TCID_50_/ml) titrated as described [84].

### Virus Purification

Rotavirus stocks were grown and purified by concentration and CsCl gradient equilibrium ultracentrifugation as described [85]. Preparations of triple-layered (TLP) and DLPs were obtained from gradients and stored at 0 ^o^C. Prior to use they were deionised using a G25 Sephadex column as described [86].

### Viral RNA Extraction

Viral dsRNA was extracted as described [84].The RNA concentration was determined by absorbance at 260 nm using a Nanodrop spectrophotometer (Nanodrop 1000, Thermo Scientific).

### Sequence Independent Amplification of Rotavirus RF Strain Genomic Segments

cDNA copies of each genome segment were amplified from viral dsRNA (extracted from purified DLPs) using the method of full-length amplification of cDNAs (FLAC) [30] with the modification of removing unincorporated anchor primer with a Qiagen RNeasy Minelute column prior to reverse transcription. The anchor primer and second primer [30] were supplied by Eurogentec S.A. RNA-anchor primer ligation products were purified by the RNeasy MinElute RNA purification column (Qiagen). cDNA and dsDNA synthesis were achieved according to [30]. Subsequently, RV ds-cDNA products were cloned into the shuttle vector TOPO TA (Invitrogen), using the manufacturer’s protocol. Clones were obtained from white colonies, amplified, screened for inserts by PCR, restriction enzyme (RE) digestion and their sequences were confirmed by Geneservice Cambridge or by GATC Biotec Cambridge using the Sanger technique with M13F, M13R and segment-specific sequencing primers. Terminal primers were designed to contain a truncated T7 Pol promoter at the 5′end of each segment and appropriate restriction enzyme recognition sites at the 3′end [20]. Appropriate RV ds-cDNAs were then subcloned into linearised plasmid pUC19. These transcription constructs were again validated by sequencing. No differences were found in comparison to the consensus sequences except in segment 7 which encodes an additional 3′ cytidine which was retained as a genetic marker (unpublished data). The addition of a single 3′ cytidine is known not to affect VP1 recognition [87]. Lists of primers used to sequence RV cDNA clones and to generate the appropriate amplicons are available upon request.

### Large Scale Amplification of Plasmids

Large cultures (300 ml) in LB broth containing either ampicillin (100 µg/ml) or kanamycin (50 µ/ml) of plasmid containing bacteria (*E. coli* DH5α) were prepared, and bacteria were harvested by low speed centrifugation. Plasmids were purified from the cell pellet using a Machery-Nagel maxi purification kit according to the manufacturer’s protocols. Dried plasmid pellets were resuspended in 100–500 µl sterile distilled water (SDW), and the plasmid concentration was determined using a Nanodrop spectrophotometer.

### *In vitro* Transcription of viral Positive sense RNAs from Digested pUC19T7 RV Templates

Firstly, plasmids were digested with either *Bsm*BI, *Bsa*I or *Bbs*I (Fermentas FastFigest®) according to the manufacturer’s instructions. To synthesise uncapped RV segmental mRNAs, 500 ng of digested plasmids (to define the 3′ end of the inserts) were used for *in vitro* transcription by the MEGAscript T7 kit (Ambion) according to the manufacturer’s protocol. For co-transcriptional capping of ssRNAs, the Mmessage Mmachine T7 kit (Ambion) was used with the following alterations: 1.5 µl of T7 RNA Pol (Ambion; 20 u/ul) per reaction was added, and the amount of linear template was increased to 1 µg for each reaction. Both un-capped and co-capped reaction mixtures were incubated at 37°C for 2.5 h. Transcripts were purified using the Qiagen Minelute RNA purification kit and quantified by Nanodrop spectrophotometry. All ssRNAs were mixed in an equal volume of Fermentas loading buffer and heated to 80°C for 10 min and cooled on ice prior to loading on agarose gels.

### Post-transciptional Cappping of ssRNAs

Purified uncapped ssRNAs synthesized *in vitro* (see above) were post-transcriptionally capped using the ScriptCap™ m7G capping system (Cambio) according to the manufacturer’s protocol. The post- transcriptionally capped ssRNAs were then purified as described above. The quality of RNA transcripts was determined by separation on 1.5% agarose gels using MOPS-EDTA formaldehyde buffer or TBE in the presence of High Range ssRNA RiboRuler™ markers (Fermentas). Purified ssRNA was stored at −70°C.

### Construction of eGFP Encoding cDNA and Reporter RNA

The eGFP ORF from plasmid pEGFP-N1 (donated by Dr Mike Gill, University of Cambridge) was cloned into pUC19 under the control of the T7 Pol promoter sequence. The eGFP PCR amplicon corresponds to the transcription cassette in the original plasmid. eGFP amplicons were synthesised using a PCR reaction with KOD DNA polymerase (Novagen) and purified using a Qiagen PCR cleaning up column (according to the manufacturer’s instructions) prior to analysis by AGE. 5 µg of PCR amplicon was digested with RE *Bsm*BI (Fermentas FastDigest®) and then purified as previously described. The digested amplicon was used for ssRNA synthesis of uncapped and co-transcriptionally capped RNAs. In addition, uncapped eGFP encoding RNA was post-transcriptionally capped *in vitro* as described above.

### Polyadenylation of ssRNAs

Both post- and co-transcriptionally capped RNAs were polyadenylated using an *E. coli* poly(A) polymerase in an Ambion polyadenylation kit according to the manufacturer’s instructions. Polyadenylated RNAs were purified and quantified as previously described and successful polyadenylation was determined by detecting a band shift using AGE.

### *In vitro* Transcription from RV DLPs

DLPs were used as particles which are transcriptionally active *in vitro* for the synthesis of ssRNAs from the encapsulated RV genomes present in the protein shells as described [34], [35], [88]). In brief, 0.5–1 ug of purified and deionised DLPs were incubated in a transcription mixture of the following composition: Tris-HCl buffer 100 mM pH 8, rNTPs 2 mM (rATP 4 mM), S-adenosyl methionine chloride (Sigma) 500 µM, MgCl_2_ 10 mM, MnCl_2_ 100 uM, DTT 5mM, RNasin (Promega) 0.2 u/µl for 3 h at 42°C. DLPs were removed by ultracentrifugation (60,000 g for 30 min), and the ssRNAs precipitated by incubation at 4°C overnight in the presence of 2M LiCl, followed by pelleting at 16,000 g for 10 min at 4°C. The ssRNA pellets were extracted twice with phenol:chloroform to remove any residual protein from degraded DLPs, precipitated and resuspended in nuclease-free water.

### Transfection Experiments

Transfection experiments were performed with nearly confluent MA104, COS-7, 293T, Vero cells in 24-, 12- or 6- well plates using Opti-MEM I medium without serum and either Lipofectamine™ 2000 (Invitrogen) or the TransIT®-mRNA transfection kit (Mirus) according to the relevant manufacturer’s instructions. Some experiments were performed on coverslips for confocal microscopy. DNA plasmids and cDNA amplicons were transfected using Lipofectamine 2000, and ssRNAs were predominantly transfected using the Mirus transfection reagent.

### Attempt for Recovery of Infectious RV from Transfected RNAs/cDNAs

Post transfection media and cell extracts were used to infect confluent monolayers of MA104 cells, as previously described [84], to check for the presence of infectious RV.

### Immunofluorescence Studies and Confocal Microscopy

Cells on coverslips were stained with specific antibodies (Table S4) prior to visualisation by either epi-fluorescence (Nikon Eclipse TE 300) or confocal microscopy (Leica TCS SP5 II).

### Sodium Dodeccyl Sulfate Polyacrylamide Gel Electrophoresis (SDS-PAGE)

SDS-PAGE was carried out as described [89].

### Western Blotting

Protein samples run using SDS-PAGE were equilibrated in transfer buffer (25 mM Tris-base, 0.2 M glycine, 20% ethanol, pH 8.3) for 15 min prior to transfer onto HiBond-C membrane (Amersham) with a Trans-Blot 3D Semi-Dry transfer cell (BioRad) at 45 mA for 45 min. Membranes were cut into appropriate horizontal strips and were blocked in 5% Milk/PBS/0.05% Tween (Sigma) overnight at 4°C. Membranes were then washed with PBS/0.05% Tween three times prior to incubation for 2 hours with appropriately diluted primary antibodies (Table S5), washed and incubated with anti-species specific secondary antibodies conjugated to horseradish peroxidase (HRP) (Table S5), for 1 hour in 1% milk/PBS. Membranes were washed prior to developing with ECL Western blotting substrate (Pierce) and exposed to X-ray film.

### *In vitro* Translation of RV Proteins from RV RNA Transcribed in vitro from cDNA Clones or Purified DLPs

Purified ssRNAs were used as templates for protein synthesis in a RRL expression system (Ambion) incorporating radiolabelled L- [^35^S]- methionine (Perkin Elmer; specific activity 1000 Ci/mM). A range of 100 ng –1 µg of ssRNA was incubated with the cell lysate according to the manufacturer’s instructions. Aminoacyl tRNAs (and template ssRNAs) were removed by digestion with 2.5 µl RNase One ribonuclease (Promega, 10 U/µl) at 30°C for 15 min. Reaction mixtures were separated by SDS-PAGE as described above. After electrophoresis proteins were fixed in [45% methanol: 10% acetic acid: SDW] for 15 min and incubated with the fluorographic reagent Amplify (Amersham) for 15 min, prior to drying for 60 min at 80°C. Dried gels were exposed to X-ray film at 4°C (Konica) for varying lengths of time. Films were developed using a Konica Minolta SRX-101A X-ray film processor.

### Intracellular Transcription of RV RNAs and eGFP Reporter RNAs

FPV recombinant expressing T7 Pol (FPV-T7) [57] was a gift from Drs Ian Goodfellow and Michael Skinner (Imperial College London). FPV-T7 was used to drive intracellular transcription in MA104, COS-7 or Caco-2 cells from cDNA amplicons encoding RV genomic segments or eGFP. Cells were infected with FPV-T7 for one hour and then transfected with the cDNAs, digested to define the 3′ end of transcription products, using Lipofectamine-2000 as previously described. Infected and transfected cells were stained with RV-specific antibodies (Table S4) to detect protein expression and visualised using either epi-fluorescence or confocal microscopy, as described above.

### Production of VLS from cDNA Clones using a FPV-T7 Recombinant

VLS [73] were created by transfecting plasmids encoding NSP2 or NSP5 into FPV-T7 infected cells. This was achieved by co-expression of NSP2 and NSP5 from pcDNA3-NSP2 or pcDNA3-NSP5 (both were gifts from Oscar Burrone, Triest, Italy). Plasmid transfection was achieved using Lipofectamine as previously described.

### DNA and Protein Sequence Analysis

The methods ClustalX2 [90] and Blast [91] were used to align cloned RV cDNA sequences and compare with other sequences, respectively. Protein sequences were analysed with Jalview [92].

## Supporting Information

Figure S1 **RV cDNA synthesis using the FLAC procedure.** Using the FLAC procedure, RV cDNA was synthesised for all 11 RV segments in a single reaction. Purified dsRNA-anchor primer hybrids were used in a self-priming RT reaction prior to amplification by PCR and a primer, 5-15-1, complementary to a region of the anchor primer sequence. Lane 1∶110 ng dsRNA, Lanes 2–4∶1:3, 1∶10 and 1∶30 dilutions of a RV cDNA, lane M: HyperLadder™ I DNA markers (in bp). Samples were analysed using a 20 mM MOPS Tris pH 7.7 AGE for 65 min at 60 V. It should be noted that segments 10 and 11 apparently comigrate in lanes 2–4; this is possibly due to migration differences of segment-anchor primer complexes. Segment 10 and 11 cDNAs were both successfully cloned from this experiment.
(TIF)

Figure S2 **T7 cassettes amplicons for each RV segment.** A clone for each RV segment was selected as the target to generate amplicons containing the T7 Pol promoter cassette amplicons. Primers were designed to specifically bind to the 5′ and 3′ termini of the particular segment of choice. Amplicons were either digested with the RE to define the 3′ end and transfected into cells as intracellular transcription templates by T7 Pol or digested with REs to facilitate cloning into pUC19. Each lane represents 5% of a PCR reaction which generated an amplicon of a RV segment. M: HyperLadder™ I DNA markers (in bp), panel A : segments 1–6; panel B: segments 7–11, respectively.
(TIF)

Figure S3 **Comparison between the RNA structures of the 5′ and 3′ termini using RNAfold.** Comparison of minimum free energy structures of RV RF strain ssRNAs using sequences obtained from GenBank and the consensus sequences derived from the FLAC cloned cDNAs. The consensus sequences were derived from the sequencing data of cDNAs introduced into the TOPO vector. The location of each 5′ and 3′ terminus is indicated, and black arrowheads indicate the location of sequencing alterations, specific details of which are found in Table S3. The colour of each base indicates the base-pairing probability as indicated by the colour scale. RNA structures were determined using RNAfold [93]. Segments 1, 6 and 9 did not encode mutations and have therefore been excluded.
(TIF)

Figure S4 **In vitro transcription and translation of segment 3 from the RV SA11 strain.** Panel A, *in vitro* transcription products from RV RF and SA11 strains using 1 µg of segment 3 cDNA *Bsm*BI digested templates. RNAs were synthesised in the presence of a cap analogue. 600 ng of ssRNA was loaded onto the gel; 1.5% TBE AGE 80 V for 45 min. Lane R: RiboRuler™ High Range; lanes 1 & 2: positive sense capped ssRNA of RV RF and SA11 strains, respectively. Panel B, *in vitro* translation of RV segment 3 ssRNAs of RF and SA11, respectively. 500 ng of co-capped ssRNA was incubated in a RRL as described, electrophoresed alongside PageRuler™ protein markers (in kDa) using 15% SDS-PAGE and exposed to X-ray film for 3 days. Lane 1: no ssRNA (negative control); lanes 2 & 3: segment 3 co-capped ssRNA of RV RF or SA11 strains, respectively. 4: XEF ssRNA (positive control).
(TIF)

Figure S5 **Polyadenylation of RV positive sense ssRNA.** Purified *in vitro* synthesised RV ssRNA was polyadenlyated in the same manner as eGFP mRNA for 1 hour at 37°C. 2% TBE AGE, 75 V for 90 min. Lane R: RiboRuler™ High Range; lanes (L) 1–8∶500 ng of ssRNAs of: L1: segment 8; L2: segment 8 polyadenylated; L3: segment 1; L4: segment 1 polyadenylated; L5: segment 9; L6: segment 9 polyadenylated; L7: segment 11; L8: segment 11 polyadenylated.
(TIF)

Figure S6 **COS-7 and MA104 cells transfection with polyadenylated RV RNAs.** MA104 (Panels A & B) and COS-7 cells (panels C & D) were fixed and stained to detect RV proteins, NSP2, NSP5 or VP1. Transfection experiments (panels A and C) were stained for NSP2, NSP5, VP1 and both NSP2 & NSP5, respectively. Panels A and C were controlled by transfection of 500 ng of eGFP mRNA yielding autofluorescence prior to staining (unpublished data). Panels B and D were infected with RV RF strain and were used a positive control. Panel B was stained for NSP2 and NSP5. Panel D was stained for VP1. Cell nuclei were stained with Hoechst 33342. Scale bars: 20 µm.
(TIF)

Figure S7 **Absence of VP2 and VP6 protein expression determined by immunofluorescence from transfected ssRNAs encoding either VP2 or VP6.** Panel A COS-7 and panel B MA104 cells at 80% confluence were transfected with ssRNAs encoding RV proteins using Mirus transfection reagent. Cells were fixed at 24 hours post transfection and stained with VP2 and VP6-specific antibodies (Table S4). Images were analysed by confocal microscopy. Cells were transfected with 1 µg of *in vitro* transcribed post-capped ssRNAs of S2, S6 or eGFP control (autofluorescencet transfection control). Immunofluorescence of COS-7 and MA104 cells infected with RF RV were stained for VP2 and VP6 (viral protein control), respectively. Cell nuclei were stained with Hoechst 33342 in all panels. Scale bars: 20 µm.
(TIF)

Figure S8 **Confirmation by Western blotting of inefficient VP2 and VP6 protein expression from transfected ssRNA.** Cells, either COS-7 (panels A and B) or MA104 (panels C and D) were transfected with 1 µg of RV ssRNA using the Mirus TransIT™ mRNA transfection reagent. Expression of RV proteins from cell lysates was sought by Western blot after separation using SDS-PAGE. The membranes, panels A and C were split into three sections to ascertain the presence of VP2, 170–70 kDa or loading control α tubulin, 70 - 40 kDa or VP6, 40–15 kDa respectively. Panels B and D, were split in two to detect α tubulin (protein loading control), 70 - 40 kDa and eGFP (transfection control) 40–15 kDa. Each membrane section was incubated with the respective primary and secondary horseradish peroxidase (HRP) conjugated antibody (Table S5). Proteins were visualised using the ECL Western blot detection reagents, light sensitive film was exposed to membranes for varying lengths of time depending on band intensity. Lane 1: mock; lanes (L) 2 - 6, *in vitro* transcribed ssRNAs or infected cell lysates. Lane 2: S2, L3:VP2, L4: S6, L5: VP6, L6: eGFP.
(TIF)

Table S1 **GenBank accession numbers for RV RF strain shotgun cloned cDNA sequences.**
(DOC)

Table S2 **GenBank accession numbers for** ***in vitro*** **transcription template consensus sequences.**
(DOC)

Table S3 **Formation of consensus sequences for each RV genomic segment.**
(DOC)

Table S4 **Primary and secondary antibodies used for confocal microscopy.**
(DOC)

Table S5 **Primary and secondary antibodies used for Western blotting.**
(DOC)
