# Supplementary figures and images for: Experimental Pathways towards Developing a Rotavirus Reverse Genetics System: Synthetic Full Length Rotavirus ssRNAs Are Neither Infectious nor Translated in Permissive Cells
Source: PLoS One. 2013 Sep 3;8(9):e74328. doi: 10.1371/journal.pone.0074328 (PMC3760874; doi:10.1371/journal.pone.0074328)

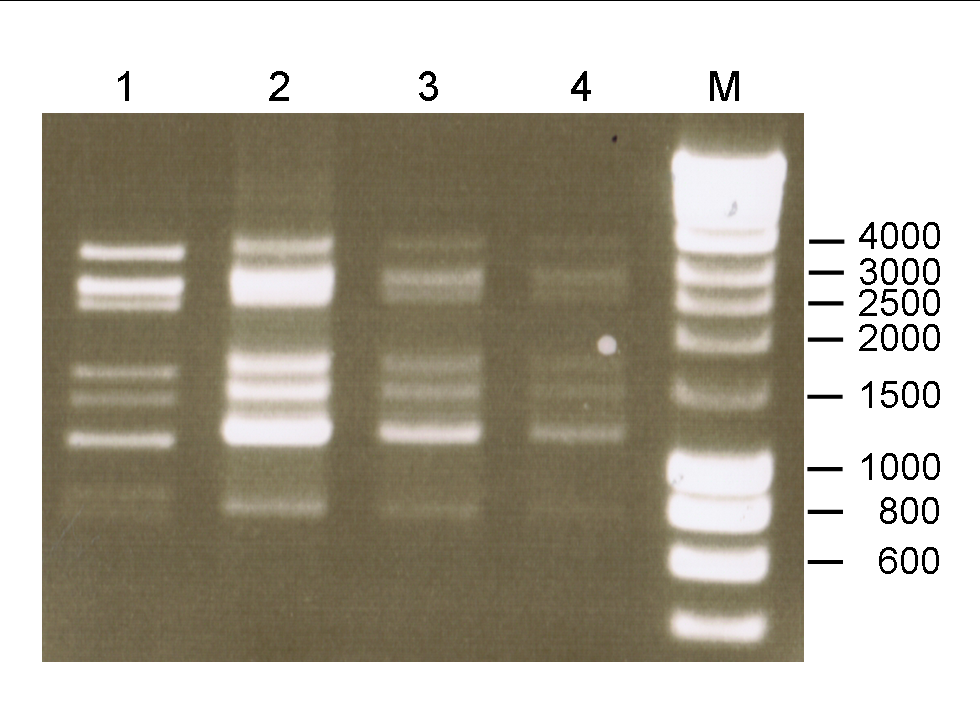

Supplement: Figure S1 — RV cDNA synthesis using the FLAC procedure. Using the FLAC procedure, RV cDNA was synthesised for all 11 RV segments in a single reaction. Purified dsRNA-anchor primer hybrids were used in a self-priming RT reaction prior to amplification by PCR and a primer, 5-15-1, complementary to a region of the anchor primer sequence. Lane 1∶110 ng dsRNA, Lanes 2–4∶1:3, 1∶10 and 1∶30 dilutions of a RV cDNA, lane M: HyperLadder™ I DNA markers (in bp). Samples were analysed using a 20 mM MOPS Tris pH 7.7 AGE for 65 min at 60 V. It should be noted that segments 10 and 11 apparently comigrate in lanes 2–4; this is possibly due to migration differences of segment-anchor primer complexes. Segment 10 and 11 cDNAs were both successfully cloned from this experiment. (TIF) [file pone.0074328.s001.tif]

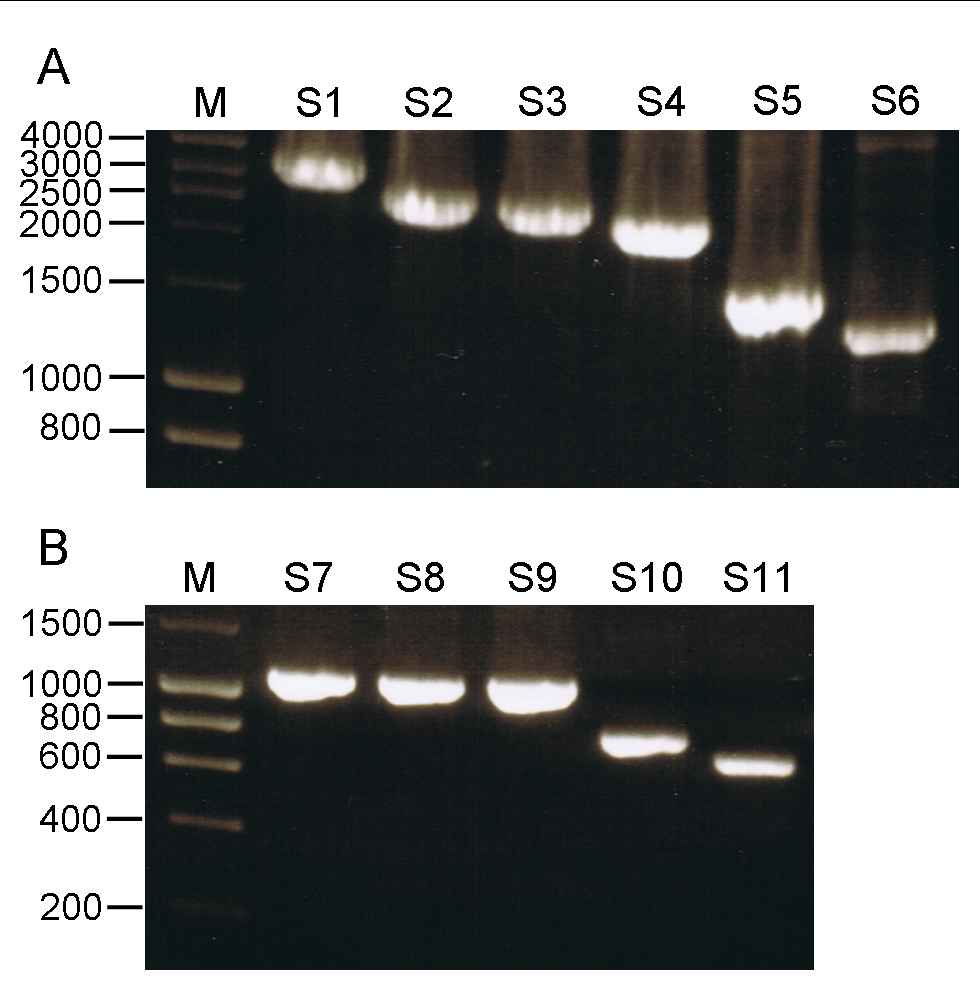

Supplement: Figure S2 — T7 cassettes amplicons for each RV segment. A clone for each RV segment was selected as the target to generate amplicons containing the T7 Pol promoter cassette amplicons. Primers were designed to specifically bind to the 5′ and 3′ termini of the particular segment of choice. Amplicons were either digested with the RE to define the 3′ end and transfected into cells as intracellular transcription templates by T7 Pol or digested with REs to facilitate cloning into pUC19. Each lane represents 5% of a PCR reaction which generated an amplicon of a RV segment. M: HyperLadder™ I DNA markers (in bp), panel A : segments 1–6; panel B: segments 7–11, respectively. (TIF) [file pone.0074328.s002.tif]

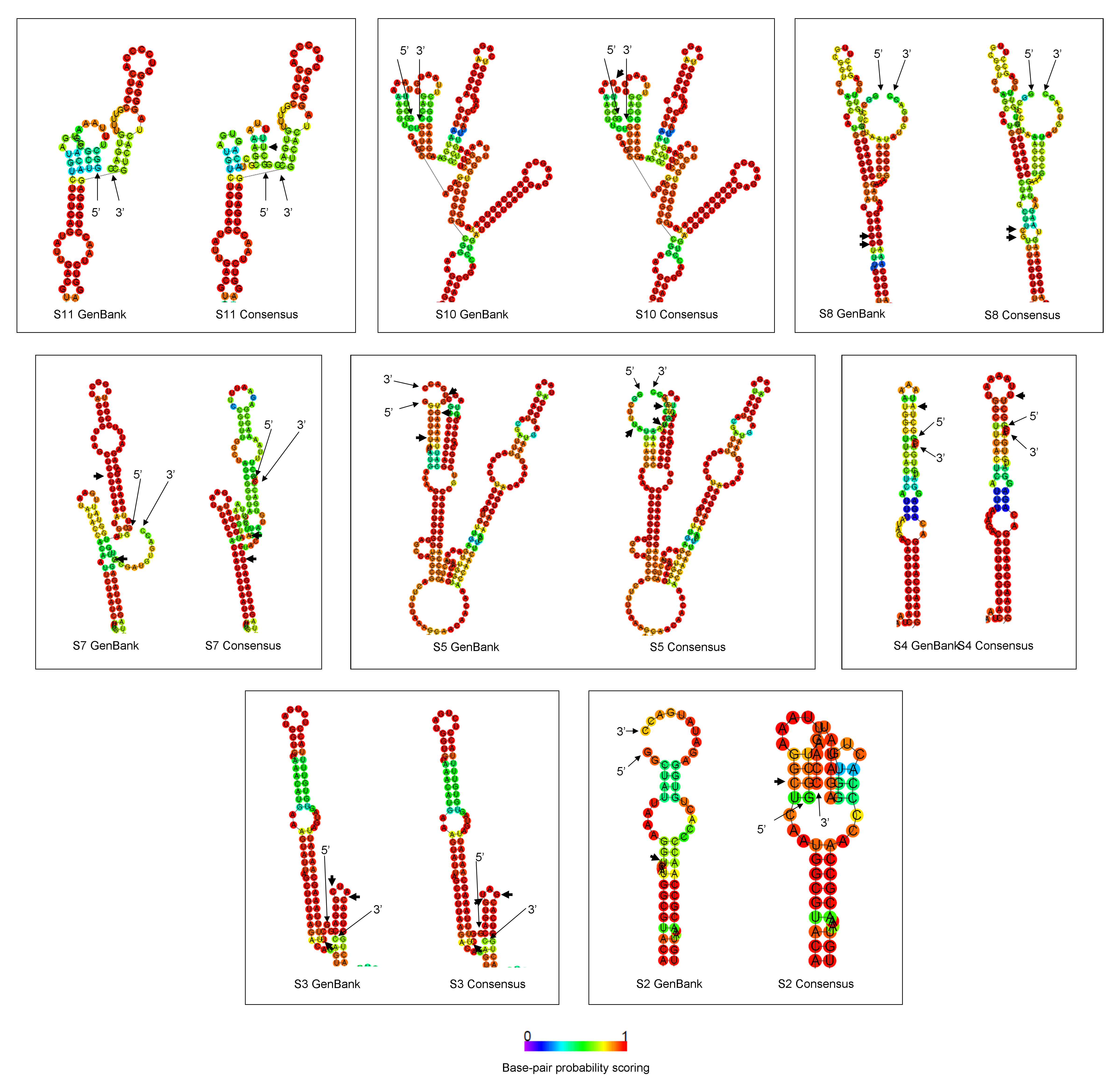

Supplement: Figure S3 — Comparison between the RNA structures of the 5′ and 3′ termini using RNAfold. Comparison of minimum free energy structures of RV RF strain ssRNAs using sequences obtained from GenBank and the consensus sequences derived from the FLAC cloned cDNAs. The consensus sequences were derived from the sequencing data of cDNAs introduced into the TOPO vector. The location of each 5′ and 3′ terminus is indicated, and black arrowheads indicate the location of sequencing alterations, specific details of which are found in Table S3. The colour of each base indicates the base-pairing probability as indicated by the colour scale. RNA structures were determined using RNAfold [93]. Segments 1, 6 and 9 did not encode mutations and have therefore been excluded. (TIF) [file pone.0074328.s003.tif]

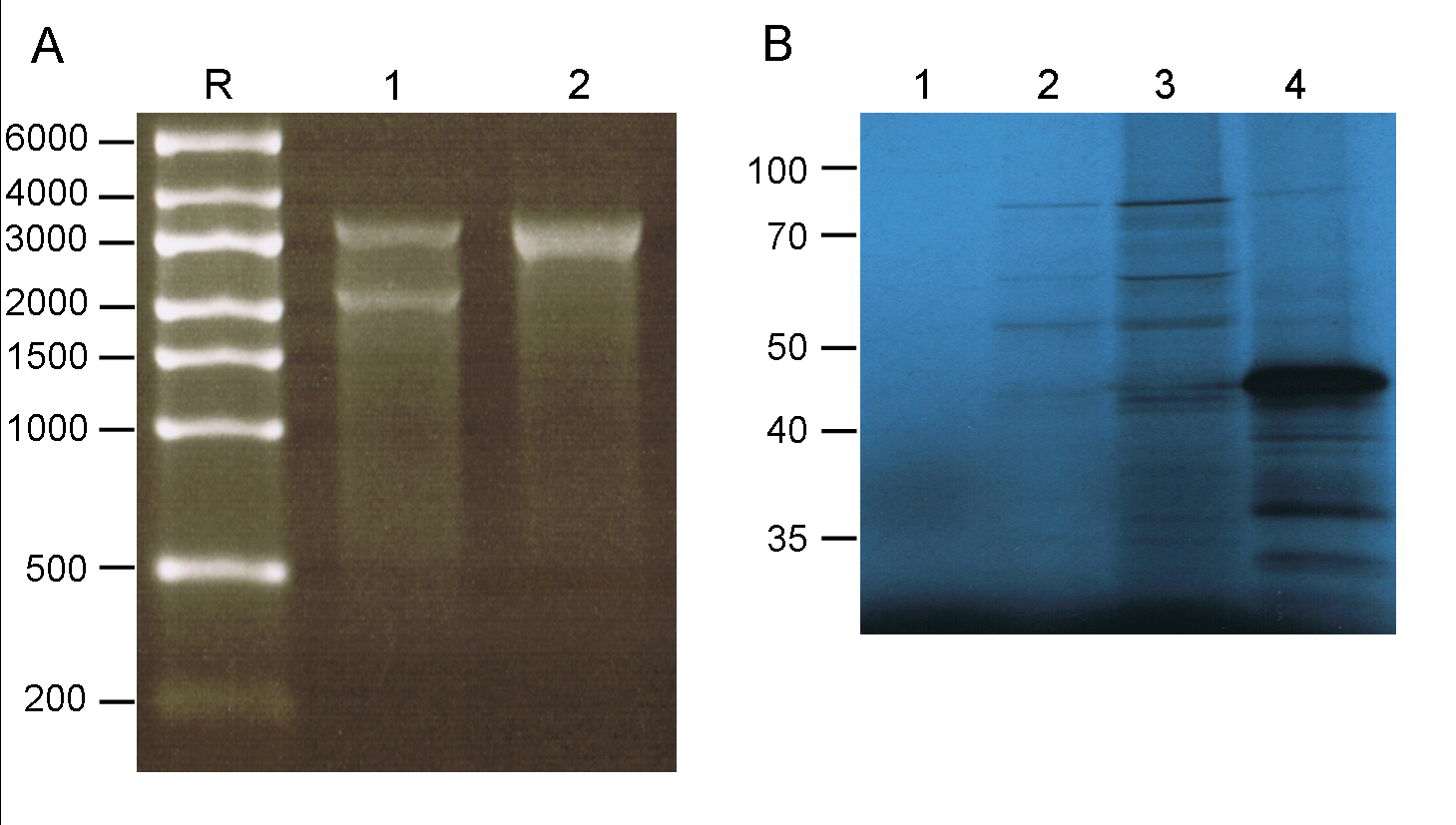

Supplement: Figure S4 — In vitro transcription and translation of segment 3 from the RV SA11 strain. Panel A, in vitro transcription products from RV RF and SA11 strains using 1 µg of segment 3 cDNA BsmBI digested templates. RNAs were synthesised in the presence of a cap analogue. 600 ng of ssRNA was loaded onto the gel; 1.5% TBE AGE 80 V for 45 min. Lane R: RiboRuler™ High Range; lanes 1 & 2: positive sense capped ssRNA of RV RF and SA11 strains, respectively. Panel B, in vitro translation of RV segment 3 ssRNAs of RF and SA11, respectively. 500 ng of co-capped ssRNA was incubated in a RRL as described, electrophoresed alongside PageRuler™ protein markers (in kDa) using 15% SDS-PAGE and exposed to X-ray film for 3 days. Lane 1: no ssRNA (negative control); lanes 2 & 3: segment 3 co-capped ssRNA of RV RF or SA11 strains, respectively. 4: XEF ssRNA (positive control). (TIF) [file pone.0074328.s004.tif]

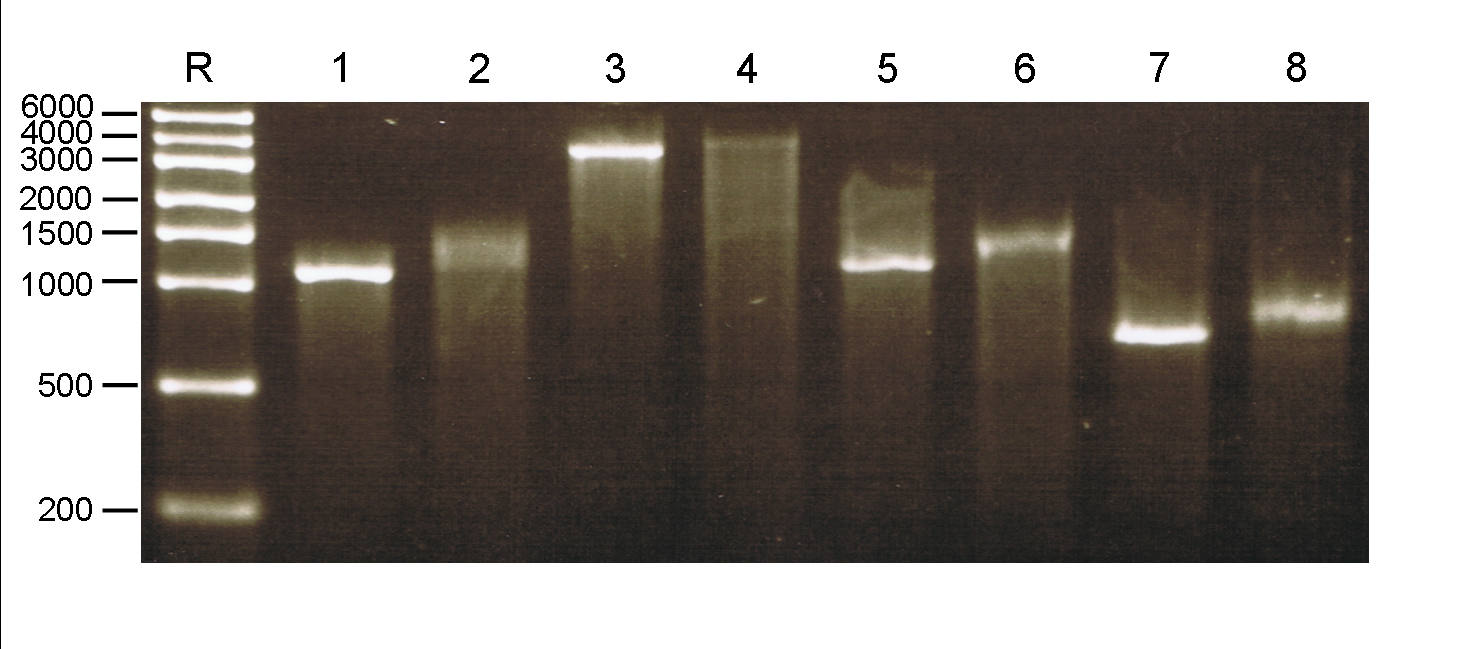

Supplement: Figure S5 — Polyadenylation of RV positive sense ssRNA. Purified in vitro synthesised RV ssRNA was polyadenlyated in the same manner as eGFP mRNA for 1 hour at 37°C. 2% TBE AGE, 75 V for 90 min. Lane R: RiboRuler™ High Range; lanes (L) 1–8∶500 ng of ssRNAs of: L1: segment 8; L2: segment 8 polyadenylated; L3: segment 1; L4: segment 1 polyadenylated; L5: segment 9; L6: segment 9 polyadenylated; L7: segment 11; L8: segment 11 polyadenylated. (TIF) [file pone.0074328.s005.tif]

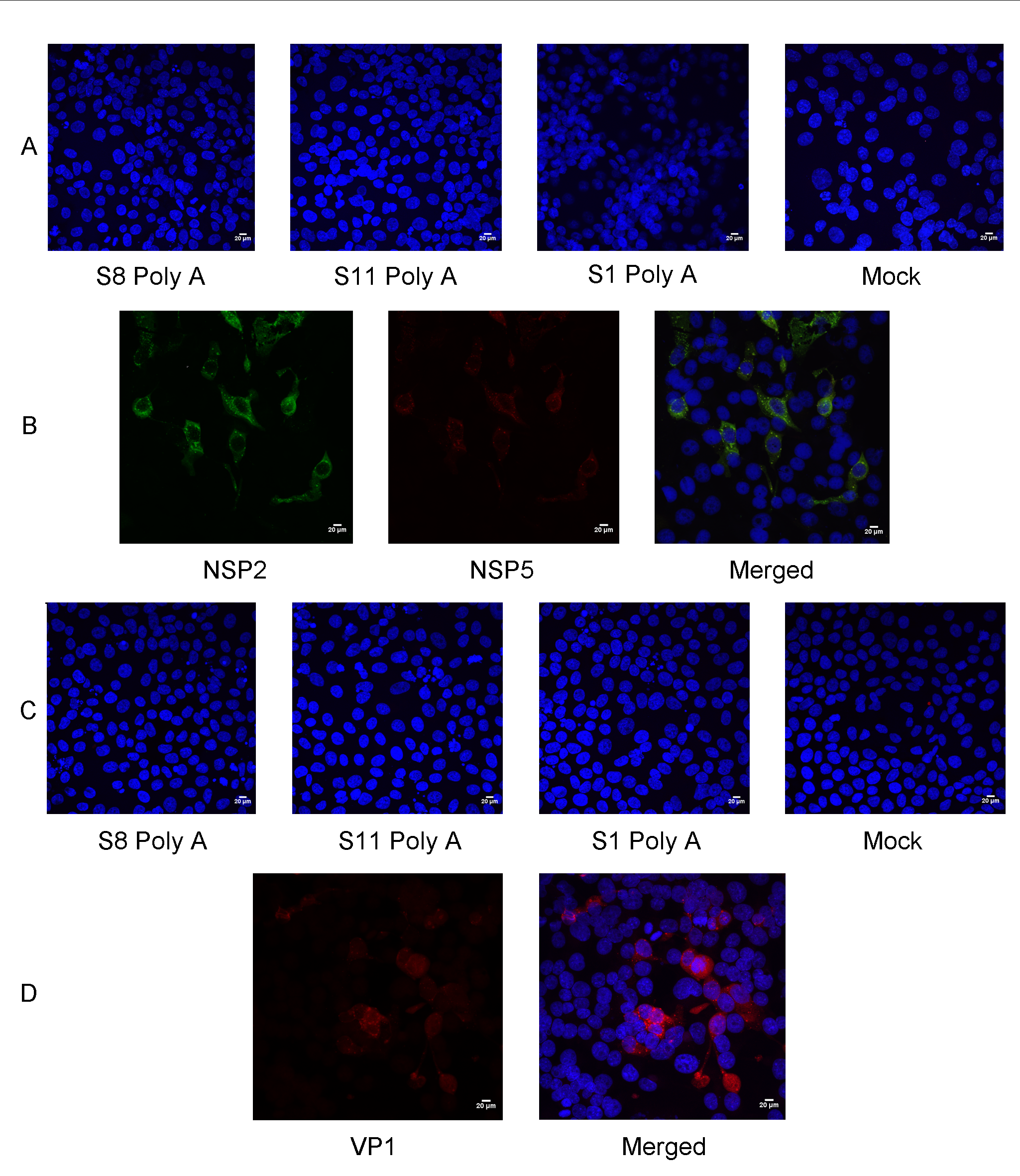

Supplement: Figure S6 — COS-7 and MA104 cells transfection with polyadenylated RV RNAs. MA104 (Panels A & B) and COS-7 cells (panels C & D) were fixed and stained to detect RV proteins, NSP2, NSP5 or VP1. Transfection experiments (panels A and C) were stained for NSP2, NSP5, VP1 and both NSP2 & NSP5, respectively. Panels A and C were controlled by transfection of 500 ng of eGFP mRNA yielding autofluorescence prior to staining (unpublished data). Panels B and D were infected with RV RF strain and were used a positive control. Panel B was stained for NSP2 and NSP5. Panel D was stained for VP1. Cell nuclei were stained with Hoechst 33342. Scale bars: 20 µm. (TIF) [file pone.0074328.s006.tif]

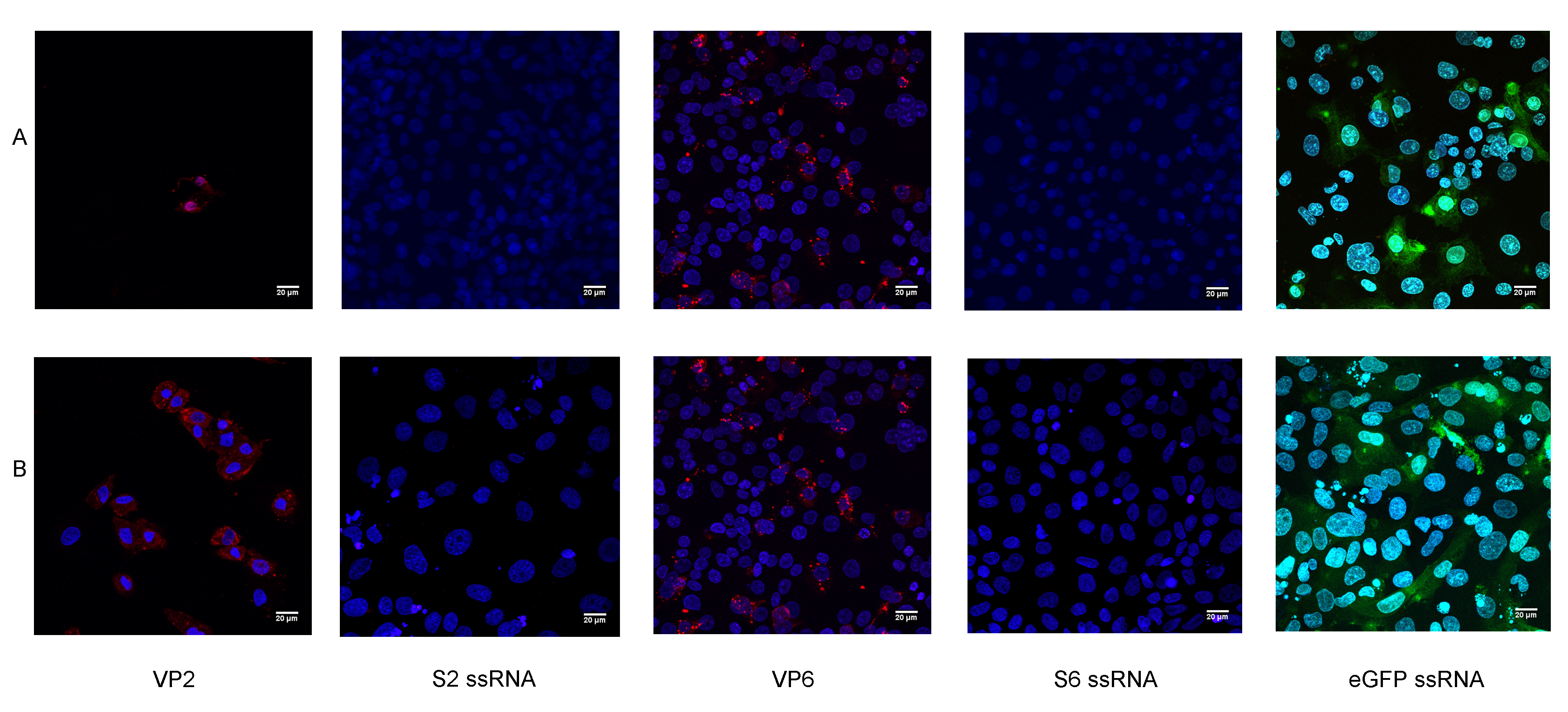

Supplement: Figure S7 — Absence of VP2 and VP6 protein expression determined by immunofluorescence from transfected ssRNAs encoding either VP2 or VP6. Panel A COS-7 and panel B MA104 cells at 80% confluence were transfected with ssRNAs encoding RV proteins using Mirus transfection reagent. Cells were fixed at 24 hours post transfection and stained with VP2 and VP6-specific antibodies (Table S4). Images were analysed by confocal microscopy. Cells were transfected with 1 µg of in vitro transcribed post-capped ssRNAs of S2, S6 or eGFP control (autofluorescencet transfection control). Immunofluorescence of COS-7 and MA104 cells infected with RF RV were stained for VP2 and VP6 (viral protein control), respectively. Cell nuclei were stained with Hoechst 33342 in all panels. Scale bars: 20 µm. (TIF) [file pone.0074328.s007.tif]

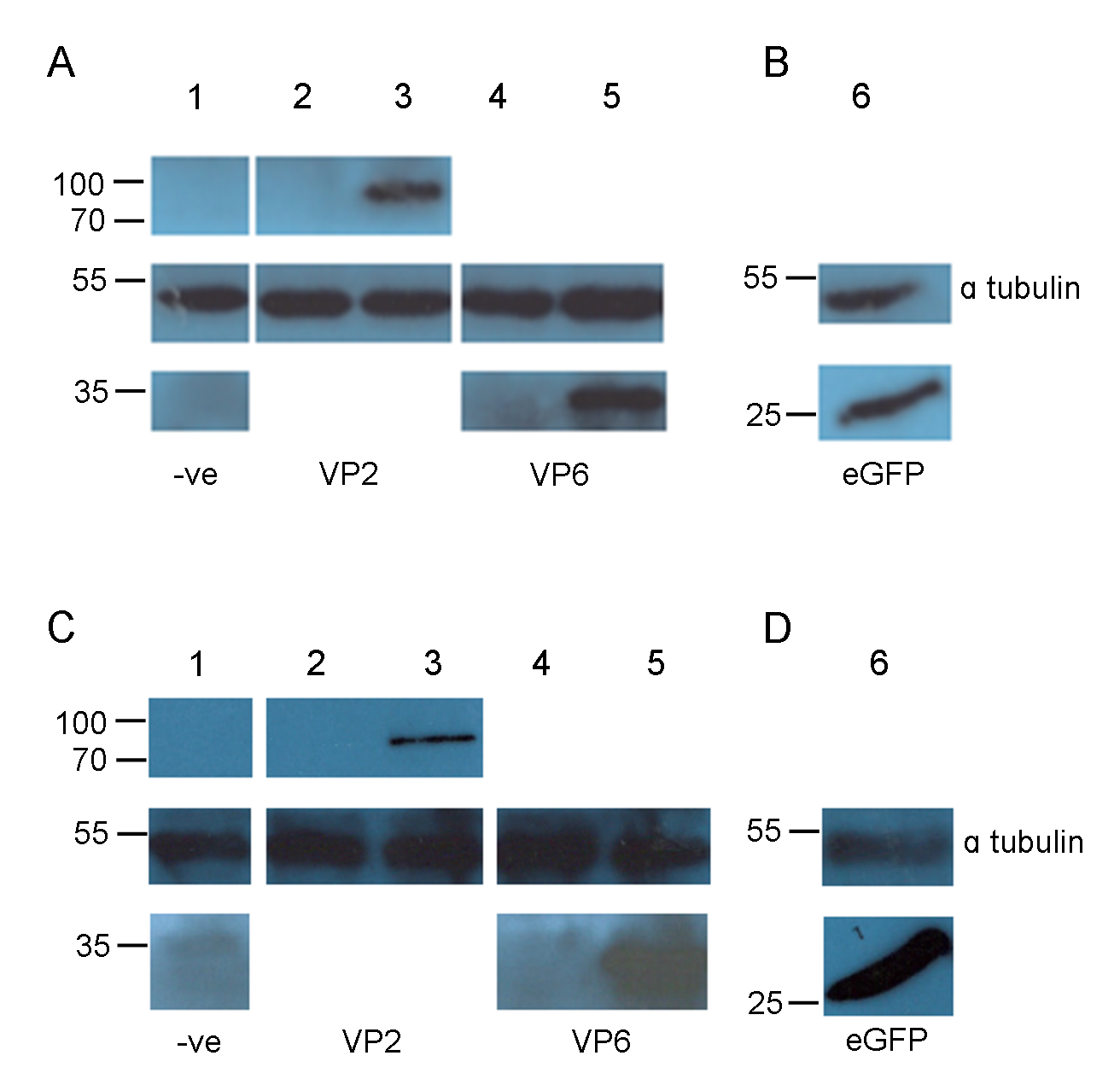

Supplement: Figure S8 — Confirmation by Western blotting of inefficient VP2 and VP6 protein expression from transfected ssRNA. Cells, either COS-7 (panels A and B) or MA104 (panels C and D) were transfected with 1 µg of RV ssRNA using the Mirus TransIT™ mRNA transfection reagent. Expression of RV proteins from cell lysates was sought by Western blot after separation using SDS-PAGE. The membranes, panels A and C were split into three sections to ascertain the presence of VP2, 170–70 kDa or loading control α tubulin, 70 - 40 kDa or VP6, 40–15 kDa respectively. Panels B and D, were split in two to detect α tubulin (protein loading control), 70 - 40 kDa and eGFP (transfection control) 40–15 kDa. Each membrane section was incubated with the respective primary and secondary horseradish peroxidase (HRP) conjugated antibody (Table S5). Proteins were visualised using the ECL Western blot detection reagents, light sensitive film was exposed to membranes for varying lengths of time depending on band intensity. Lane 1: mock; lanes (L) 2 - 6, in vitro transcribed ssRNAs or infected cell lysates. Lane 2: S2, L3:VP2, L4: S6, L5: VP6, L6: eGFP. (TIF) [file pone.0074328.s008.tif]
