# Supplementary material for: Experimental Pathways towards Developing a Rotavirus Reverse Genetics System: Synthetic Full Length Rotavirus ssRNAs Are Neither Infectious nor Translated in Permissive Cells
Source: PLoS One. 2013 Sep 3;8(9):e74328. doi: 10.1371/journal.pone.0074328 (PMC3760874; doi:10.1371/journal.pone.0074328)
Supplement: Table S1 — GenBank accession numbers for RV RF strain shotgun cloned cDNA sequences. (DOC) [file pone.0074328.s009.doc]

**Table S1. GenBank accession numbers for RV RF strain shotgun cloned cDNA sequences.**

| **Construct Name** | **GenBank Accession Number** |
| --- | --- |
| TopoRF1#1 | Tbc |
| TopoRF1#2 | Tbc |
| TopoRF1#3 | Tbc |
| TopoRF1#4 | Tbc |
| TopoRF1#5 | Tbc |
| TopoRF2#1 | Tbc |
| TopoRF2#2 | Tbc |
| TopoRF2#3 | Tbc |
| TopoRF2#4 | Tbc |
| TopoRF3#1 | Tbc |
| TopoRF3#2 | Tbc |
| TopoRF3#3 | Tbc |
| TopoRF3#4 | Tbc |
| TopoRF3#5 | Tbc |
| TopoRF4#1 | Tbc |
| TopoRF4#2 | Tbc |
| TopoRF4#3 | Tbc |
| TopoRF4#4 | Tbc |
| TopoRF4#5 | Tbc |
| TopoRF5#1 | Tbc |
| TopoRF5#1 | Tbc |
| TopoRF5#2 | Tbc |
| TopoRF5#3 | Tbc |
| TopoRF5#4 | Tbc |
| TopoRF5#5 | Tbc |
| TopoRF6#1 | Tbc |
| TopoRF7#1 | Tbc |
| TopoRF7#2 | Tbc |
| TopoRF7#3 | Tbc |
| TopoRF7#4 | Tbc |
| TopoRF7#5 | Tbc |
| TopoRF7#6 | Tbc |
| TopoRF8#1 | Tbc |
| TopoRF8#2 | Tbc |
| TopoRF8#3 | Tbc |
| TopoRF8#4 | Tbc |
| TopoRF9#1 | Tbc |
| TopoRF9#2 | Tbc |
| TopoRF9#3 | Tbc |
| TopoRF9#4 | Tbc |
| TopoRF9#5 | Tbc |
| TopoRF9#6 | Tbc |
| TopoRF9#7 | Tbc |
| TopoRF9#8 | Tbc |
| TopoRF10#1 | Tbc |
| TopoRF11#1 | Tbc |
| TopoRF11#2 | Tbc |
| TopoRF11#3 | Tbc |
| TopoRF11#4 | Tbc |
| TopoRF11#5 | Tbc |
| TopoRF11#6 | Tbc |
| TopoRF11#7 | Tbc |
| TopoRF11#8 | Tbc |
| TopoRF11#9 | Tbc |
