# Supplementary material for: Experimental Pathways towards Developing a Rotavirus Reverse Genetics System: Synthetic Full Length Rotavirus ssRNAs Are Neither Infectious nor Translated in Permissive Cells
Source: PLoS One. 2013 Sep 3;8(9):e74328. doi: 10.1371/journal.pone.0074328 (PMC3760874; doi:10.1371/journal.pone.0074328)
Supplement: Table S2 — GenBank accession numbers for in vitro transcription template consensus sequences. (DOC) [file pone.0074328.s010.doc]

**Table S2. GenBank accession numbers for *in vitro* transcription template consensus sequences.**

| **Construct Notation** | **GenBank Accession number** |
| --- | --- |
| puc19T7S1wt | Tbc |
| puc19T7S2Mt | Tbc |
| puc19T7S3Mt | Tbc |
| puc19T7S4Mt | Tbc |
| puc19T75Mt | Tbc |
| puc19T7S6wt | Tbc |
| puc19T7S7Mt | Tbc |
| puc19T7S8Mt | Tbc |
| puc19T7S9wt | Tbc |
| puc19T7S10Mt | Tbc |
| puc19T7S11Mt | Tbc |
