# Supplementary material for: Experimental Pathways towards Developing a Rotavirus Reverse Genetics System: Synthetic Full Length Rotavirus ssRNAs Are Neither Infectious nor Translated in Permissive Cells
Source: PLoS One. 2013 Sep 3;8(9):e74328. doi: 10.1371/journal.pone.0074328 (PMC3760874; doi:10.1371/journal.pone.0074328)
Supplement: Table S3 — Formation of consensus sequences for each RV genomic segment. (DOC) [file pone.0074328.s011.doc]

**Table S3. Formation of consensus sequences for each RV genomic segment**

| **Consensus sequence construct notation** | **Consensus sequence length (nucleotides)** | **RV**  **protein** | **Number of cDNA clones used to derive consensus sequence** | **GenBank Accession of genomic sequence aligned to cDNA sequences** | **Clone specific mutations (Nucleotide number - N#)** | **Isolate specific mutations**  **(Nucleotide number - N#)** | **Effect on translated protein sequence**  **(AA# - amino acid number)** | |
| --- | --- | --- | --- | --- | --- | --- | --- | --- |
| **Segment 1**  **puc19T7RFS1wt** | 3302 | VP1 | 5 | J04346 | Clone #3; N#1 & N#2; G deletion  N#188 A - G point mutation  Clone #5;  N#2616 T - C | None | Clone #3; AA# 57K – R  Other nucleotide mutation are synonymous or outside of the ORF | |
| **Segment 2**  **puc19T7RFS2Mt** | 2687 | VP2 | 4 | X1057 | Clone #3;  N#157 C - A N #2687 C deletion  Clone #4; N# 1378 A – G | 4/4 clones have point mutations:  N#13 T - C  N#1377 G - C  N#1896 G - C  N#2347 C – G  N#2348 G - C  N#2572 T - A | Common mutations in ORF  AA# 454 R - T AA# 627 G - A  AA# 777 N - K  AA# 778 V – L  Clone #4; 219 G - stop  Other nucleotide mutation are synonymous or outside of the ORF | |
| **Segment 3**  **puc19T7RFS3Mt** | 2593 | VP3 | 5 | AYY116592 | Clone #4;  N#4 T - C  Clone #5;  N#1 G deletion | 5/5 clones have  N#8 T deletion  N#2586 G insertion  N#2588 C deletion | All nucleotide mutation are synonymous or outside of the ORF | |
| **Segment 4**  **puc19T7RFS4Mt** | 2362 | VP4 | 5 | U65924 | 4/5 clones N#1 G deletion (not clone #1)  5/5 clones N#5 A - T | Clone #1;  N#1646 C –A N#1810 A - G  Clone #3;  N#521 G – A  Clone #4; N#2 G deletion  Clone #5; N#2 G deletion  N#389 A - G N#1089 A - G N#2089 A - G | | Clone #1; AA 546 T - K AA#601 I – V  Clone #2 ; AA#171 G- E  Clone #5; AA#127 Q - R AA#694 K – E  Other nucleotide mutation are synonymous or outside of the ORF |
| **Segment 5**  **puc19T7S5Mt** | 1579 | NSP1 | 5 | M22308 | 5/5 clones have a T deletion at N#7 and N#1569.  5/5 clones have a G deletion at N#1574 | Point mutations:  Clone #2; N#531 G - A  Clone #4; N#1496 A - G | | Clone #2; AA#167 E – K  Clone #4; silent mutations  Other nucleotide mutation are synonymous or outside of the ORF |
| **Segment 6**  **puc19T7RFS6wt** | 1356 | VP6 | 1 | K02254 | None | None | | None |
| **Segment 7**  **puc19T7S7Mt** | 1062 | VP7 | 6 | X65940 | 6/6 clones have point mutations;  N#43 C - A,  N#195 A – T,  N#997 C – T,  N#1048 G - T | Clone #2; T deletion at N#278  Point mutations:  Clone #4; N#189 A - G,  Clone #5; N#139 G - A and N#1062 C deletion | | Common mutations in ORF;  AA#49 K - N and AA# 317 L - F  Clone #2; AA#77 L - stop codon due to T deletion at N#278  Clone #4; AA#47 I - M  Clone #5; AA#31 D – N  Other nucleotide mutation are synonymous or outside of the ORF |
| **Segment 8**  **puc19T7S8Mt** | 1059 | NSP2 | 4 | Z21640 | 4/4 clones have point mutations;  CG-GC at N# 93 & 94 | Clone #4; N#572 C - T | | Common mutation:  4/4 clones AA#16 S – T  Other nucleotide mutation are synonymous or outside of the ORF |
| **Segment 9**  **puc19T7S9wt** | 1074 | NSP3 | 8 | Z21639 | No common mutations | Clone 1 has a T insertion at N#4  Point mutation:  Clone #2; N#568 T - C  Clone #4: N#599 A deletion and point mutations: N#645 T - C and N#808 T - C  Point mutations:  Clone #5; N#829 C - A  Clone #7; N#893 A – G | Clone #2; AA# 181 F - S  Clone #4; at AA#101, 11 mutations then a stop codon due to point mutation N#599  Clone #5; AA#268 S - Y  Clone #7; AA# 289 I - M  Other nucleotide mutation are synonymous or outside of the ORF | |
| **Segment 10**  **puc19T7S10Mt** | 751 | NSP4 | 1 | AY116593 | Clone #1 has point mutation;  N#7 T - A | Point mutation:  Clone #1; N#7 T - A | All nucleotide mutations are synonymous or outside of the ORF | |
| **Segment 11***  **puc19T7S11Mt** | 667 | NSP5 and NSP6 | 9 | AF188126 | 9/9 clones; N#4 T insert | Clone #3; N#1 G deletion  Point mutations:  Clone #8; N#2458 C - A  Clone #9 N#475 G - A | Clone #3; none NSP5 or NSP6  Clone #8; AA#74 S – stop codon due to point mutation N#245 in NSP5  Clone #9; aa 153 A – T in NSP5  Other nucleotide mutation are synonymous or outside of the ORF | |

The GenBank and cDNA sequences were aligned using ClustalX2 and analysed in Jalview. The impact of nucleotide mutation was examined and mutations in the ORFs of viral proteins were compared to that of wild type proteins (GenBank accession number provided).

*Segment 11; NSP5 opening reading frame N#21 - 617, NSP6 open reading frame N#79 - 375.
