# Supplementary material for: Experimental Pathways towards Developing a Rotavirus Reverse Genetics System: Synthetic Full Length Rotavirus ssRNAs Are Neither Infectious nor Translated in Permissive Cells
Source: PLoS One. 2013 Sep 3;8(9):e74328. doi: 10.1371/journal.pone.0074328 (PMC3760874; doi:10.1371/journal.pone.0074328)
Supplement: Table S4 — Primary and secondary antibodies used for confocal microscopy. (DOC) [file pone.0074328.s012.doc]

**Table S4. Primary and secondary antibodies used for confocal microscopy.**

| **Protein raised against** | **Species raised in** | **Conjugate** | **Dilution used** | **Source** |
| --- | --- | --- | --- | --- |
| NSP2 | Mouse | N/A | 1:500 | Oscar Burrone |
| NSP5 | Guinea pig | N/A | 1:500 | Oscar Burrone |
| VP1 | Guinea pig | N/A | 1:500 | John Patton |
| VP2 | Guinea pig | N/A | 1:500 | John Patton |
| VP6 | Guinea pig | N/A | 1:500 | John Patton |
| eGFP | Goat | N/A | 1:500 | Abcam (AB5450) |
| anti-mouse | Goat | Alexa Fluor® 488 | 1:500 | Invitrogen (A11029) |
| anti-guinea pig | Goat | Texas Red | 1:300 | Santa Cruz (SC-2441) |
| anti-goat | Rabbit | Alexa Fluro® 568 | 1:500 | invitrogen (A11079) |
| anti-mouse | Goat | Alexa Fluro® 568 | 1:500 | invitrogen (A11031) |
