# Supplementary material for: Experimental Pathways towards Developing a Rotavirus Reverse Genetics System: Synthetic Full Length Rotavirus ssRNAs Are Neither Infectious nor Translated in Permissive Cells
Source: PLoS One. 2013 Sep 3;8(9):e74328. doi: 10.1371/journal.pone.0074328 (PMC3760874; doi:10.1371/journal.pone.0074328)
Supplement: Table S5 — Primary and secondary antibodies used for Western blotting. (DOC) [file pone.0074328.s013.doc]

**Table S5. Primary and secondary antibodies used for Western blotting.**

| **Protein raised against** | **Species raised in** | **Conjugate** | **Dilution used** | **Source** |
| --- | --- | --- | --- | --- |
| NSP5 | Guinea pig | N/A | 1:500 | Oscar Burrone |
| VP1 | Guinea pig | N/A | 1:500 | John Patton |
| VP2 | Guinea pig | N/A | 1:500 | John Patton |
| VP6 | Guinea pig | N/A | 1:500 | John Patton |
| α-tubulin | Rabbit | N/A | 1:500 | Abcam (AB4074) |
| eGFP | Goat | N/A | 1:500 | Abcam (AB5450) |
| anti-guinea pig | Rabbit | HRP | 1:500 | Dako (P0141) |
| anti-rabbit | Goat | HRP | 1:500 | Dako (P0448) |
| anti-goat | Rabbit | HRP | 1:500 | Dako (P0160) |
